# Liposomal Formulations for an Efficient Encapsulation of Epigallocatechin-3-Gallate: An In-Silico/Experimental Approach

**DOI:** 10.3390/molecules23020441

**Published:** 2018-02-16

**Authors:** Emiliano Laudadio, Cristina Minnelli, Adolfo Amici, Luca Massaccesi, Giovanna Mobbili, Roberta Galeazzi

**Affiliations:** 1 Dipartimento di Scienze della Vita e dell’Ambiente (DISVA), Università Politecnica delle Marche, via Brecce Bianche, 60131 Ancona, Italy; e.laudadio@univpm.it (E.L.); c.minnelli@pm.univpm.it (C.M.); luca.massaccesi@gmail.com (L.M.); 2 Dipartimento Scienze Cliniche Specialistiche ed Odontostomatologiche, Università Politecnica delle Marche, via Brecce Bianche, 60131 Ancona, Italy; a.amici@univpm.it

**Keywords:** epigallocathechin gallate, liposomes, lipid bilayer simulations

## Abstract

As a part of research project aimed to optimize antioxidant delivery, here we studied the influence of both salts and lipid matrix composition on the interaction of epigallocatechin-3-gallate (EGCG) with bilayer leaflets. Thus, we combined in silico and experimental methods to study the ability of neutral and anionic vesicles to encapsulate EGCG in the presence of Ca^2+^ and Mg^2+^ divalent salts. Experimental and in silico results show a very high correlation, thus confirming the efficiency of the developed methodology. In particular, we found out that the presence of calcium ions hinders the insertion of EGCG in the liposome bilayer in both neutral and anionic systems. On the contrary, the presence of MgCl_2_ improves the insertion degree of EGCG molecules respect to the liposomes without divalent salts. The best and most efficient salt concentration is that corresponding to a 5:1 molar ratio between Mg^2+^ and EGCG, in both neutral and anionic vesicles. Concerning the lipid matrix composition, the anionic one results in better promotion of the catechin insertion within the bilayer since experimentally we achieved 100% EGCG encapsulation in the lipid carrier in the presence of a 5:1 molar ratio of magnesium. Thus, the combination of this anionic liposomal formulation with magnesium chloride, avoids time-consuming separation steps of unentrapped active principle and appears particularly suitable for EGCG delivery applications.

## 1. Introduction

Nanostructures made of amphiphilic natural or synthetic lipids are biocompatible and biodegradable platforms for drug delivery [1,2,3,4,5,6,7,8,9]. Among them, liposomes have emerged as highly promising self-assembled nanovectors able to entrap both lipophilic and hydrophilic agents in the lipid membrane and in the aqueous core, respectively [10]. Liposomes present some important advantages: for example, they can increase the stability of the encapsulated molecules and improve their pharmacokinetic effect by reducing elimination and enhancing circulation lifetimes. Epigallocatechin-3-gallate (EGCG) is the major constituent in green tea [11]; it presents remarkable bioactivity ranging from anticancer [12,13] to cancer- and in general age-associated pathology prevention effects [14,15] as well as regulation of the immune [16] and endocrine systems [17].

Despite its high activity, the use of EGCG as a drug or natural additive is compromised by its oxidizability and instability that determine a poor bioavailability within the biological system whatever the administration mode is [18,19,20].

**Figure 1:**
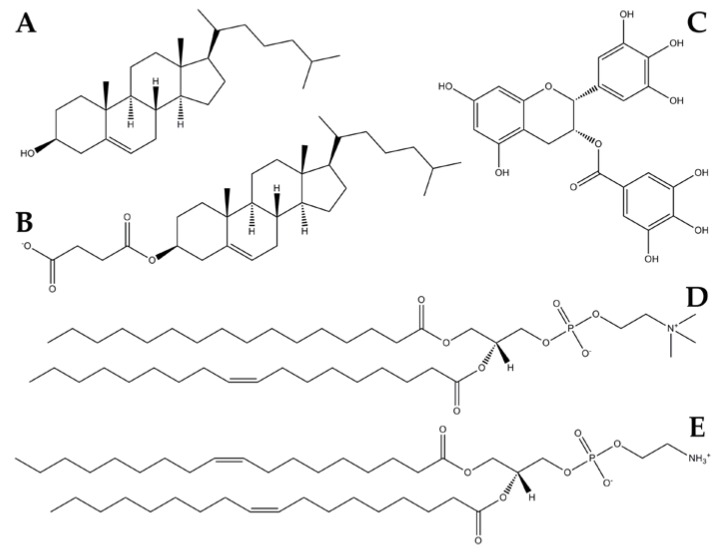
2D structures of cholesterol (Chol) (**A**), cholesteryl hemisuccinate (CHEMS) (**B**), 1-palmitoyl-2-oleoylphosphatidyl-choline (POPC) (**C**), Epigallocatechin-3-gallate (EGCG) (**D**) and 1,2-Dioleoyl-sn-glycero-3-phosphoethanolamine (DOPE) (**E**).

In addition, the presence of numerous phenolic groups (Figure 1) makes the molecule susceptible to various metabolic reactions such as sulfonidation, glucuronidation and methylation leading to an inadequate pharmacokinetic profile [21] when administered orally. The introduction of EGCG inside liposomes could protect the molecule from degradation, thus increasing its concentration and absorption at the target site; for this purpose it is crucial to maximize the encapsulation percentage in order to optimize the delivery of this active principle.

Moreover, it is well known that the interaction between metal cations (particularly Mg^2+^ and Ca^2+^) and lipids plays an essential role in the structure and function of biological membranes. In particular, many studies have paid special attention to the ability of Ca^2+^ or Mg^2+^ to induce aggregation and fusion of liposomes with the cellular membrane, thus improving the penetration of the drugs carried by the liposomes inside the cells [22,23,24,25].

In a previous work [26], we already used an in silico approach to study the influence of salt addition in EGCG and morelloflavone (MF) interaction with 1-palmitoyl-2-oleoylphosphatidyl-choline (POPC) liposomes. Starting from these results, in this work we focused on the influence of lipid matrix composition on promoting EGCG interaction at the bilayer surface. Thus, we combined our in silico protocol and experimental methods to study the ability of neutral and anionic vesicles to entrap EGCG in the presence of divalent Ca^2+^ and Mg^2+^ salts; our final aim is to find out optimal conditions to obtain the maximum percentage of encapsulated catechin.

## 2. Results and Discussion

### 2.1. Effect of Salts and Lipid Composition on EGCG Encapsulation Efficiency Inside Multilamellar Vesicles (MLVs)

We mixed POPC, the most common unsaturated phosphatidylcholine (PC) in eukaryotic membranes, with DOPE (1,2-Dioleoyl-sn-glycero-3-phosphoethanolamine) to take advantage of its fusogenic properties in prevision of an EGCG delivery application; moreover we added cholesterol (Chol) and cholesteryl hemisuccinate (CHEMS) to obtain neutral and negative self-assembling lipid systems, respectively (Figure 1). 

Neutral and anionic MLVs, containing 460 μg/mL of EGCG, were prepared with an equimolar lipid ratio of POPC/DOPE/Chol and POPC/DOPE/CHEMS, respectively, and a final lipid concentration of 3 mg/mL. 

In addition, the presence of the cholesterol skeleton can contribute to the bilayer stability guaranteeing a reproducible formulation for pharmaceutical applications [27]. The choice of EGCG amount is crucial—too high concentration of EGCG can alter and finally disrupt the membrane structure [28]. Because of its water solubility, EGCG tends to diffuse into the aqueous phase during the emulsion process; in order to improve the encapsulation efficiency it is consequently important to employ liposome formulations able to enhance the interaction of the molecule with the lipid matrix. Starting from our previous results [26], we proceeded to investigate the effect of divalent salts on the encapsulation efficiency in these new liposomal vectors, employing increasing quantities of MgCl_2_ and CaCl_2_ (1:1, 3:1, 5:1 and 6:1 salt and EGCG molar ratio) that can interact not only with neutral and charged phospholipids by Coulombic forces but also with the phenolic and aromatic groups of EGCG. Our experimental results suggest the existence of substantial differences in the Ca^2+^ and Mg^2+^ interaction with lipids and EGCGs at level of the molecular scale organization of the lipid bilayer (Table 1).

We obtained the best result with the anionic system (POPC/DOPE CHEMS) and Mg^2+^ ions; in fact, by increasing the concentration of magnesium salt, we observe an enhancement in EGCG encapsulation with a maximum of efficiency in correspondence of a magnesium/EGCG molar ratio of 5:1 when upon we obtained a practically complete encapsulation. It worth pointing out that in order to ensure that a salts’ effect is not to cause precipitation of the EGCG, we centrifuged control solutions containing EGCG alone in aqueous suspension mixed with calcium or magnesium chloride at the same concentrations used for the liposomal formulations. These controls did not show any sign of EGCG precipitation. The polyphenol OD (λ = 280 nm) after centrifugation did not show any sensible decrease with respect to the corresponding reference solutions also using the same speed and conditions selected for sedimentation of liposomes (see Supplementary Material). With any further increase of the Mg^2+^ concentration (6:1, magnesium/EGCG molar ratio), the percentage of antioxidant molecules inside the liposomes decreases by about 20%. Magnesium chloride improves the encapsulation efficiency inside neutral liposomes too, but without reaching the very high value of the anionic system. Also with neutral MLVs the optimal ratio EGCG: Mg^2+^ is 5:1. Besides, the calcium salt is not able to optimize the efficiency of encapsulation neither in anionic nor in neutral systems. On the contrary, the calcium presence seems to prevent the insertion of EGCG in the liposome bilayer and we obtained the worst results with anionic liposomes where the encapsulation efficiency is drastically reduced respect to the system without a bivalent salt (Table 1). We also observed that the presence of Ca^2+^ and Mg^2+^ induces strong aggregation of liposomes (0.5–5 μm) but we have obtained optimal results in the prevention of this phenomenon by adding a biocompatible stabilizing agent like Tween-20. We chose Tween-20 due to its low cost, biodegradability and biocompatibility [29,30] and the tendency of non-ionic surfactant molecules to promote the formation of stable liposomes [31]. In order to proceed with future drug delivery uses, we focused only on the liposomal formulation that achieved 100% encapsulation efficiency. After addition of Tween-20 at a concentration of 0.28 mM we obtained vesicles of about 340 nm that maintain the high encapsulation efficiency (EE) (96.2 ± 3.8 %) and remain stable after 24 h (Supplementary Materials). In conclusion, we can achieve a high EE by using a favorable concentration of salt and contrast its aggregating effect using a stabilizing agent.

With the aim to better understand and rationalize the EGCGs encapsulation efficacy inside anionic and neutral liposomes in presence of Ca^2+^ and Mg^2+^ divalent salts, we next carried out MD simulations that can shed light on the molecular organization of our lipid bilayers and on the interactions occurring between EGCGs and the bilayer surface.

### 2.2. Interaction of EGCG with Lipidic Bilayer: Simulation Results

#### 2.2.1. Neutral Lipid Models

We built neutral lipid bilayer models considering that EGCG aggregates obtained from a preliminary 70 ns MD simulation in pure water reported in our previous work [26] (see Supplementary Material). Each aggregate is formed by five EGCG molecules and its average dimensions are X 20.54, Y 16.92, Z 28.05 Å. We used such an EGCG configuration for our 200 ns lipid bilayer MD simulations. More precisely, this EGCG aggregate was put in the aqueous phase at a distance from phosphate groups ranging from 10 to 25 Å.

**Figure 2:**
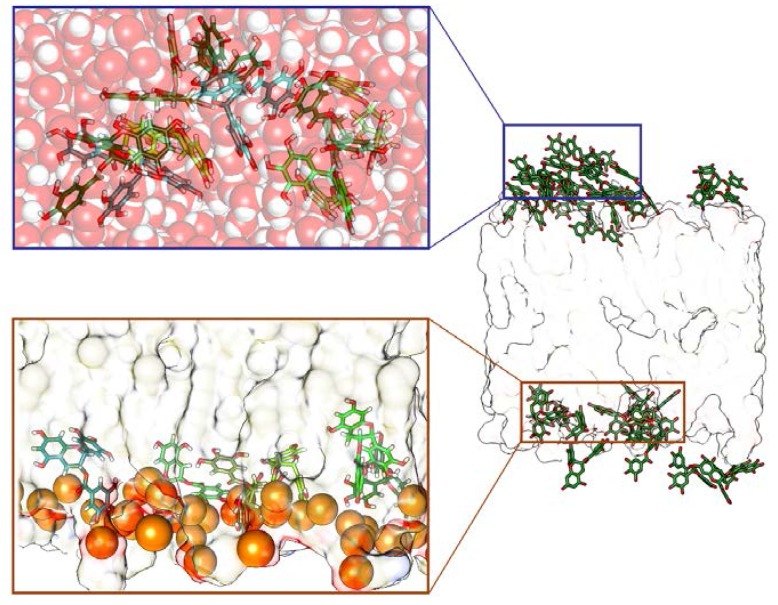
Neutral lipid system in absence of divalent salts: Epigallocatechin-3-gallate (EGCG) molecules (as green tube models), and the lipid leaflets (as transparent surface) are shown. The permanence of the EGCGs aggregate during the interactions with the bilayer can be observed.

In the absence of divalent salts, at the end of MD simulations, we obtained very stable models, with part of the EGCG molecules still involved in the intramolecular aggregate (Figure 2) [26]. Consequently, we did not observe efficient EGCGs insertion within the membrane, but instead an adhesion onto the bilayer surface of the cathechins in their aggregate form. Only the few molecules moved away from the initial intermolecular aggregate, and interact efficiently with the neutral lipid bilayer’s phosphates (Figure 2).

In CaCl_2_ neutral bilayer models, by increasing the calcium chloride concentration, all the simulated models reached the steady state after 70 ns (Supplementary Material Table S1).

**Figure 3:**
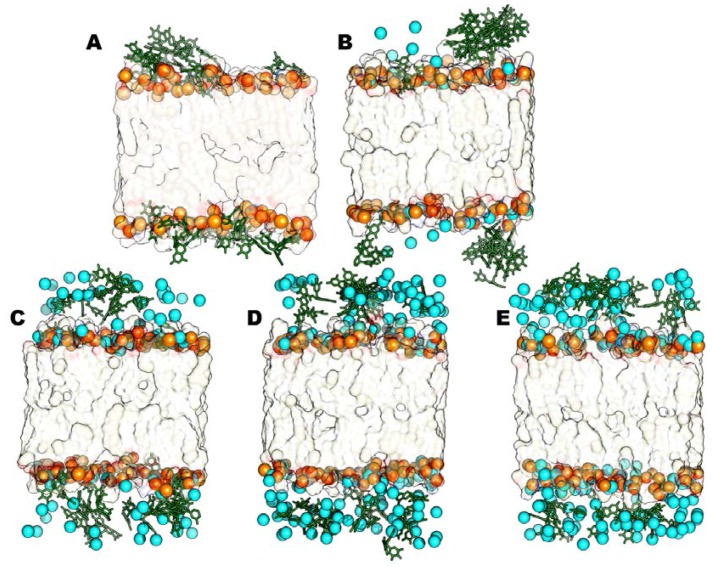
Representative structures for the steady states of neutral models with CaCl_2_. The models without salts (**A**), 1:1 (**B**), 3:1 (**C**), 5:1 (**D**) and 6:1 (**E**) are shown. Ca^2+^ ions (cyan VdW spheres), Epigallocatechin-3-gallate (EGCG) molecules (green sticks), phosphorous atoms (orange VdW spheres) and lipid leaflets (as transparent surface) are highlighted.

Analyzing representative structures of all the CaCl_2_ models (Figure 3), we observe that the increasing concentrations of calcium chloride promotes the interaction of Ca^2+^ ions with DOPE and POPC’ phosphates, thus preventing the catechins’ insertion at the bilayer surface. In fact, calcium ions are able to break their solvation shell and locate themselves near phosphate groups, competing with EGCG molecules in creating very strong ionic interactions with the lipids’ head groups.

**Figure 4:**
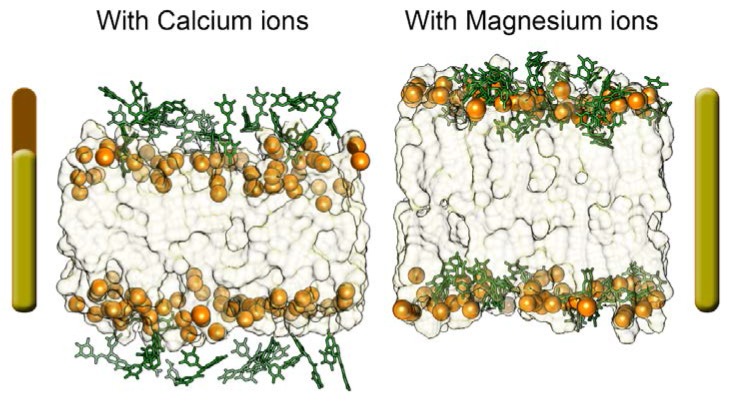
Comparison of bilayer dimension along Z-axis between (5:1) salt: Epigallocatechin-3-gallate (EGCG) neutral models (CaCl_2_ model left, MagCl_2_ model right).

**Figure 5:**
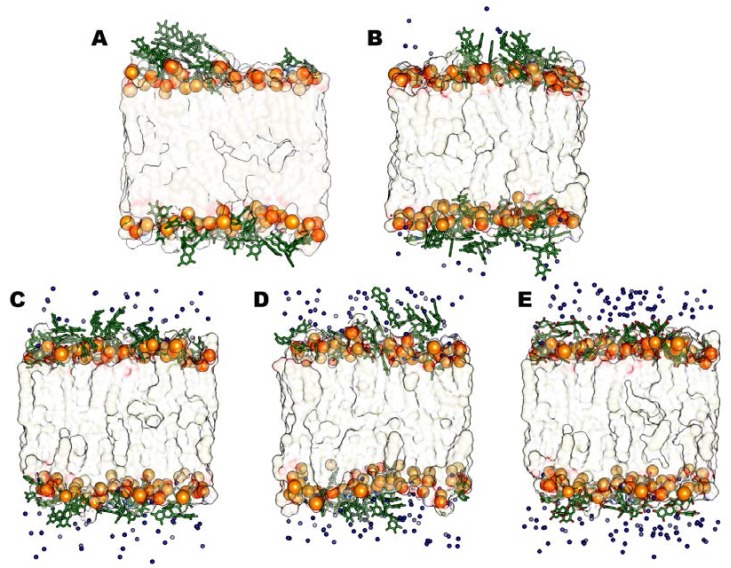
Representative structures for the steady states of neutral models with MgCl_2_. The models without salts (**A**), 1:1 (**B**), 3:1 (**C**), 5:1 (**D**) and 6:1 (**E**) are shown. Mg^2+^ ions (blue VdW spheres), Epigallocatechin-3-gallate (EGCG) molecules (green sticks), phosphorous atoms (orange VdW spheres) and lipid leaflets (as transparent surface) are highlighted.

Therefore EGCGs stay more in the aqueous phase respect to the system where divalent ions are absent; the ECGC penetration degree is very low, and it is even less with an increasing CaCl_2_ concentration, in particular in the 6:1 system, where the catechin molecules are clearly far from the phosphates. In addition, most of them are involved in cation-π interactions with the calcium ions present in the water phase. Then, we repeated the same experiments described above for CaCl_2_ but changing the salt. We added MgCl_2_ in the same concentrations used for the CaCl_2_ models (1:1, 3:1, 5:1 and 6:1, salt and EGCC molar ratio). From the corresponding MD trajectories analysis, we observed that all the four simulated systems reached stability very early, precisely after 40 ns of simulation. It is interesting to observe that Mg^2+^ lies in solution more than Ca^2+^ and thus it does not compete with EGCG in interacting with the lipids’ phosphates. Beside, due to its high positive charge density, magnesium ions electrostatically attract lipids’ polar phosphates, thus promoting a stretching of the bilayer that becomes more extended towards the bilayer Z-axis than the other models do. This is particularly evident from the comparison of the final representative bilayer structures (Figure 4) and from membrane thickness values (Table 2). This structural feature appears to improve the penetration of EGCG molecules that are less fixed at the bilayer surface. Finally, we did not find any differences on the EGCGs’ behavior after increasing the MgCl_2_ concentration (Figure 5). Indeed, some peculiar influence of magnesium cation in lipid bilayer organization has also been observed in other mixed composition models [22,25,26,32,33].

#### 2.2.2. Anionic Lipid Models

We applied the same computational protocol to build the corresponding anionic lipid bilayers, substituting Chol molecules with CHEMS. The latter is a cholesterol synthetic derivative with a negative net charge (Figure 1); in this way, we built the anionic mixed composition bilayer, containing POPC, DOPE and CHEMS compounds in 1:1:1 molar ratio respectively. Once again, we considered the corresponding model without the divalent salt and besides we simulated the bilayer anionic models containing CaCl_2_ and MgCl_2_ in the same molar ratios reported for the neutral lipid ones.

At the end of the MD simulations, we found that all systems reached stability, as observed for the previous neutral models. We analyzed the EGCG behavior in the anionic model (no divalent salt), and we observed an increase of the catechins’ insertion within the leaflets with respect to the corresponding neutral system analyzed in the absence of divalent salt.

**Figure 6:**
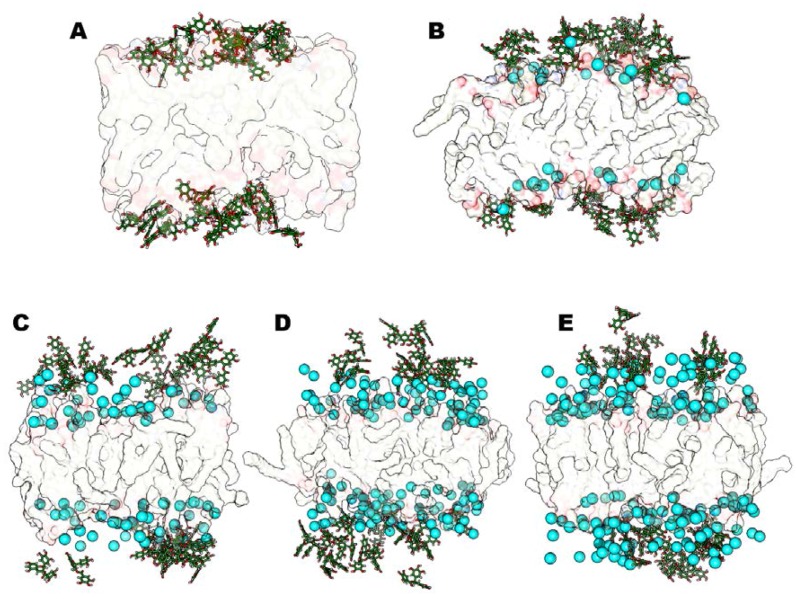
Representative structures for the steady states of anionic systems with CaCl_2_. The models without salts (**A**), 1:1 (**B**), 3:1 (**C**), 5:1 (**D**) and 6:1 (**E**) are shown. Ca^2+^ ions (cyan VdW spheres), Epigallocatechin-3-gallate (EGCG) molecules (green sticks), and lipid leaflets (as transparent surface) are highlighted.

In this case, the aggregation effect between catechins that we observed in the neutral control system (Figure 2) is not so evident, and a majority of the EGCG molecules interact efficiently with the anionic lipids’ phosphates (Figure 6A).

As for the neutral models, we focused on the effect of calcium chloride; we noted that after increasing CaCl_2_ concentration we did not observe differences in the EGCG behavior: in fact, the catechin remains in water phase in its aggregate form and does not interact with the lipids’ polar heads.

Also in this case, all four simulated systems reach stability after 40 ns of MD simulation and we did not observe any system modification between the four studied systems. During the production phase of the MD simulation, all EGCG molecules keep on lying in solvent medium, more than in the absence of salt, and this does not depend on the amount of CaCl_2_ that we introduced. For this reason, we observe similar fluctuations of EGCG molecules along all MD simulations (Figure 6).

This phenomenon is due to the strong interaction of Ca^2+^ ions with the anionic CHEMS molecules, and the negatively charged phosphate groups of DOPE and POPC. The calcium ions, as described above, break the solvation shell and array themselves very close to the bilayer polar headgroups, and this behaviour is more evident when the CaCl_2_ concentration increases. The strong interactions between divalent cations and CHEMS molecules did not allow the EGCG molecules to interact with bilayer, even in the model with a 1:1 molar ratio. Finally, we considered bilayers containing MgCl_2_ salt in the described negative charged lipid environment. The MD simulations results confirmed our previously reported results [26]: MgCl_2_ can extensively improve the EGCG interactions with the bilayer lipids where CaCl_2_ cannot.

**Figure 7:**
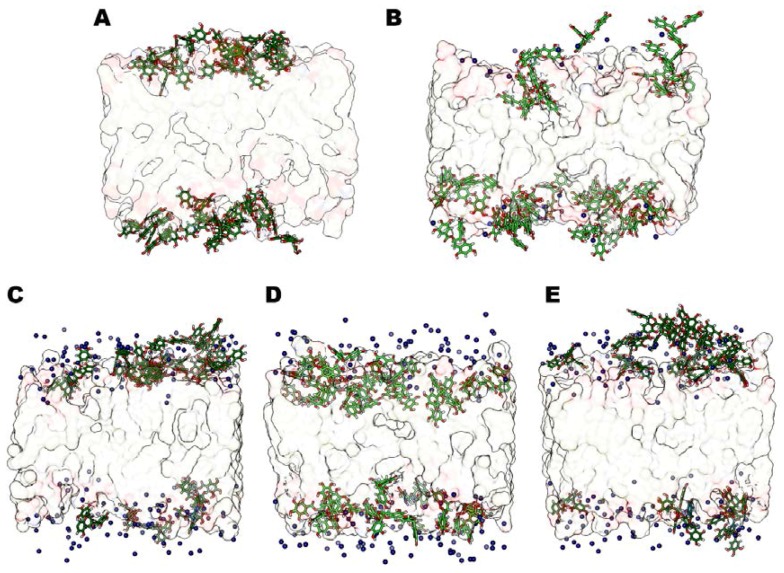
Representative structures for the steady states of anionic systems with MgCl_2_. The models without salts (**A**), 1:1 (**B**), 3:1 (**C**), 5:1 (**D**) and 6:1 (**E**) are shown. Mg^2+^ ions (blue VdW spheres), Epigallocatechin-3-gallate (EGCG) molecules (green sticks), and lipid leaflets (as transparent surface) are highlighted. The model D shows that all EGCG molecules are inserted inside the membrane.

In fact, also in these anionic systems, magnesium ions interact with phospholipids but lie in solvent medium and do not compete with catechin molecules for phosphate lipids’ interaction. Therefore, in the presence of MgCl_2_ we always observe an evident increase of EGCG penetration. In addition, we found that the systems reached stability very early, during the first 25–35 ns of MD simulations, and they have all very similar stabilization pathways, except for the system with MgCl_2_ in 5:1 molar ratio with EGCG molecules that reaches stability at 80 ns of MD simulation. We can explain this totally different trend by a major structural transformation of the system. The analysis of some representative structures of all simulated models (Figure 7) points out that the 5:1 MgCl_2_-EGCG model appears very different from the others.

In fact, this model (5:1) shows all catechin molecules inserted in the anionic bilayer leaflets (Figure 7D). The higher amount of time that is necessary to reach the steady state correlates with a major reorientation needed for EGCG molecules, which here all interact with the membrane. This behavior is once again mediated by Mg^2+^ ions lying in the water phase promoting the membrane stretching, thus increasing membrane thickness (Table 2) [22,25,26]. Besides, we can also observe an increase of the average Area per lipid (Areapl) values that corresponds to a major cathechin insertion inside the leaflets. In the 5:1 model, we determined the optimal salt concentration that maximizes the penetration of EGCG molecules. In the 6:1 model, the catechin’s insertion degree is in line with 1:1 and 3:1 systems, and since the Mg^2+^ ions concentration is very high, part of magnesium ions directly interact with CHEMS compounds and POPC and DOPE polar headgroups, competing with catechin compounds and thus decreasing the degree of EGCG insertion within the bilayer surface compared to the 5:1 model. All these experimental findings are in line with the reported experimental results and can explain the different trend of encapsulation in different vectors correlating them with the amount of EGCGs interactions and insertion within the bilayer leaflets (Table 1).

#### 2.2.3. Time-Averaged Distributions of EGCG, Mg^2+^/Ca^2+^

**Figure 8:**
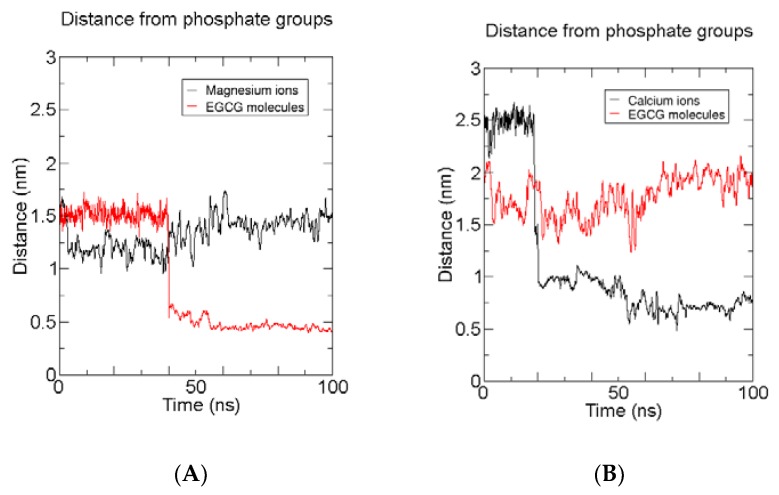
Distance evolution through MD trajectory of cations and Epigallocatechin-3-gallate molecules (EGCGs) form the bilayer surface (lipid phosphates) for MgCl_2_: EGCG 5:1 (**A**) and CaCl_2_:EGCG 6:1 models (**B**). For purpose of clarity, only the first 100 ns of the trajectories are reported.

In order to better put into evidence the salt interactions responsible for the different trend of EGCG insertion in presence of magnesium and calcium salts, we calculated the time-averaged distribution for EGCGs and the cations with respect to the bilayer surface in the best performant model (anionic 5:1 MgCl_2_:EGCG) and in the analogue system with calcium (anionic 5:1 CaCl_2_:EGCG). The results obtained are reported in Figure 8.

Indeed, it is clear that the two models show a completely opposite trend. In fact, for the magnesium model, while the Mg^2+^ ions kept their position in solution through all the simulation, EGCGs molecules after about 40 ns move on the bilayer surface and continue with their insertion inside the leaflets. On the contrary, in the calcium chloride anionic model, EGCGs remain in solution while Ca^2+^ ions only after 20 ns shift to the bilayer surface.

In order to identify which bilayer components better interact with EGCG, we analyzed the distance of EGCGs from the phosphate groups of POPC and DOPE, and from succinate moiety of CHEMS for the MgCl_2_:EGCG 5:1 anionic model which is the most efficient system.

**Figure 9:**
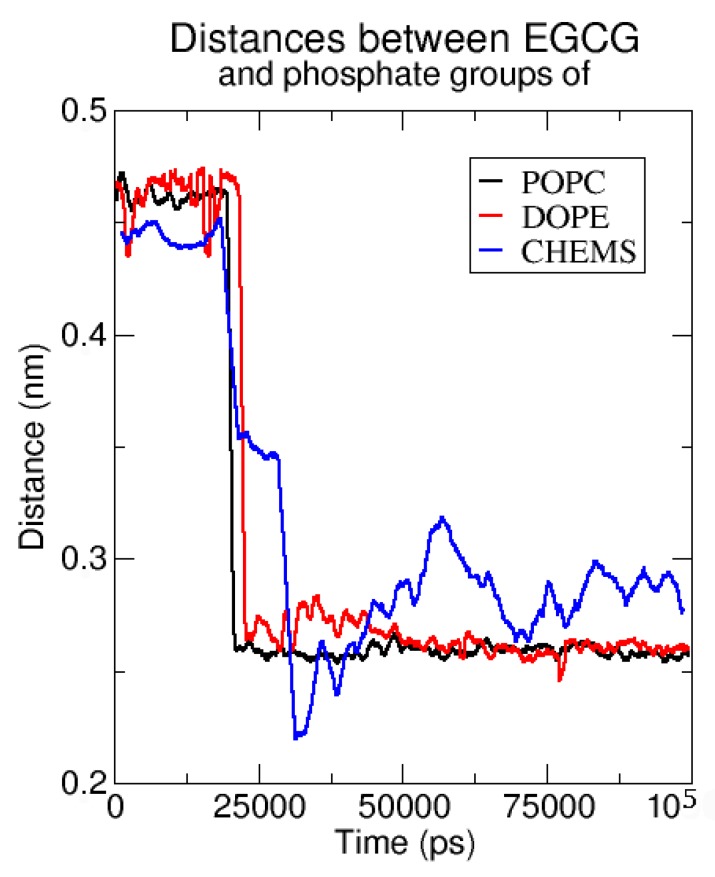
Distance of Epigallocatechin-3-gallate molecules (EGCGs) from phophate groups of 1-palmitoyl-2-oleoylphosphatidyl-choline (POPC) and 1,2-Dioleoyl-sn-glycero-3-phosphoethanolamine (DOPE), and from the succinate moiety of cholesteryl hemisuccinate (CHEMS) for the 5:1 MgCl_2_:EGCG anionic model.

This can shed light on what triggers EGCG ad- and absorption by the membrane. From Figure 9, we can see that the catechins interact more strongly with POPC and DOPE phosphates as can be deduced from the value of the distance of EGCG from those groups that is constant starting from 40–50 ns of the trajectory (Figure 9). On the contrary, the oscillatory values of the EGCG distance from CHEMS’ succinate moieties suggest that the latter component is freer to move due to a minor amount of direct interactions with EGCGs. Anyway the presence of the negative charged steroid CHEMS increases the electrostatic interaction with the magnesium cations; thus, it indirectly can promote the catechin insertion, thus explaining why the anionic model is the best one.

## 3. Materials and Methods

### 3.1. Computational Methods

#### 3.1.1. Parameterization of Epigallocatechin-3-Gallate and CHEMS

Epigallocatechin-3-gallate (EGCG) and cholesteryl hemisuccinate (CHEMS) molecules were firstly parametrized following the same protocol previously reported [26]. Thus, we explored their conformational potential energy surface (PES) in order to localize the main stationary points, i.e., lowest energy minimum and the most populated conformers. Then, molecular mechanics energy calculations were performed using the AMBER force field implemented in the Maestro/MacroModel (Schrodinger Inc., Portland, OR, USA) software framework [34], and the torsional space of the molecules was randomly varied with the usage-directed Monte Carlo Multiple Minimum (MCMM) conformational search approach [35]. For each search, 1000 starting structures for each variable torsion angle were generated and then minimized until the gradient was less than 0.05 kJ Å^−1^ mol^−1^. We used quantum mechanical calculations (DFT, B3LYP/6-311G**) to calculate EGCG and CHEMS’s Mulliken charges. The solvent effect was included by using the implicit water GB/SA solvation method [36], and duplicate conformations and those with an energy in excess of 6.0 kcal mol^−1^ above the global minimum were discarded. The cluster analysis was performed within the Macromodel package using X-cluster following a protocol already reported [37,38,39,40,41,42]. Then, for each compound (EGCG and CHEMS), we carried out the clustering considering the RMSD of the main carbons skeleton (threshold value RMSD = 0.5) as filter. We found out for both molecules one most populated cluster (about 90–95%) that corresponds also to the global minimum’s cluster. The resulting lowest energy conformers, together with the representative structures of the most populated clusters, were optimized and the charges re-calculated by DFT calculation using G09 suite of Gaussian 09, Revision D.01 software [43] at B3LYP/6-311G** level of theory [44,45,46,47,48], in order to better take into account the electronic effects in the conformer’s stabilization and populations [49]. For the lowest energy conformers of EGCG and CHEMS, we then fitted the missing CHARMM parameters using the VMD toolkit/G09 [50] and we added the corresponding topology file using Xleap tool [51]. For POPC, DOPE (1,2-dioleoyl-sn-glycero-3-phosphoethanolamine), Chol (Cholesterol), TIP3P water molecules we used the already included CHARMM parametrization.

#### 3.1.2. Molecular Dynamics of EGCG and in Mixed Lipid Bilayers

All the molecular dynamics (MD) simulations of the mixed lipid bilayer were carried out on the isothermal–isobaric (N, P, T) ensemble at 1 atm and 310 K (37 °C). The GROMACS 5.0.4 suite of programs [52] was used with CHARMM [44] force field [33] parameter sets, using EGCG and CHEMS’s charges and parameters previously calculated [45,46,47,48,53]. This force field results particularly accurate for lipid bilayer dynamics [53,54]. The membrane leaflets are composed by a total of 144 lipid molecules hydrated by 4758 water molecules within an initial simulation box (corresponding to a pre-equilibrated mixed bilayer) that was 8 nm (Z) normal to the bilayer and 7 nm long in each of the two dimensions of the bilayer plane (XY). We started from neutral mixed composition lipid system (POPC, DOPE and Cholesterol in molar ratio 1:1:1 between them) was obtained by membrane builder tool of CHARMM-GUI website (www.charmm-gui.org) with an extension of 10 ns to equilibrate systems by NPT ensemble simulation [53]. To generate the anionic mixed lipid model, we substituted all Cholesterol molecules with CHEMS compounds. We added 30 EGCG molecules at every mixed composition bilayer considered in this study, corresponding to the EGCG molar concentration used experimentally. In our previous published work [26], we carried out MD simulation putting 30 molecules of the catechin in water, starting from a casual and not ordered molecules’ orientation. As a result, we found out the formation of a stable EGCG’s aggregate (Supplementary Material). Mixed composition bilayers considered in this study were built adding the antioxidant molecules in the same configuration as found out in the equilibrated solvated model. All the simulations were conducted in presence of divalent metal salts CaCl_2_ and MgCl_2_ at different concentrations (1:1, 3:1, 5:1 and 6:1 in molar ratio between salt and EGCG). The TIP3P model for solvent has been used and ions were added to reach salt concentrations. Water and ions overlapping the membrane bilayer were removed before proceeding to system minimization [55]. The chosen system dimensions were based on literature reports concerning the smallest representative size that can be used to accurately reproduce the occurring intermolecular interactions in lipid bilayers [56,57,58]. In particular, MD simulations have been extensively carried out by Klauda and co-workers on different sized systems (72 up to 288 lipids) to examine system size dependence on dynamical properties associated with the Particle Mesh Ewald (PME) treatment of electrostatic interactions. Each bilayer system was energy minimized under periodic box conditions (re-modulated starting cell unit in nm, X = 7.00, Y = 7.00, Z = 8.00) applied in all directions using a neighbour searching grid type, and also setting at 1.4 nm the cut-off distance for the short range neighbour list. The Verlet cut-off scheme was used for neighbour searching, combined with PME for electrostatics. Cut-off for the calculation of Van der Waals forces was set to 1.2 nm, with the force smoothly switched to zero between 1.0 and 1.2 nm. Electrostatic were taken into account implementing a fast smooth particle-mesh Ewald (SPME) algorithm [59,60,61,62] with a 1.4 nm distance for the Coulomb cut-off, since this method is considered to be both efficient and accurate for the evaluation of long-range electrostatic interactions in large macromolecular systems [37,38,39]. For all the MD trajectories, we used the NPT ensemble maintaining the weak coupling also for pressure control (i.e., Berendsen barostat). Velocities were first generated at 310 K in the NVT ensemble, using a Maxwell distribution function with random seed; a weak temperature coupling (Berendsen thermostat), with time constant of 1 ps, was applied to maintain the reference temperature (310 K) for the whole run; no water was observed inside the bilayer. At this point a 200 ns dynamics has been set up for each of the built system. An accurate leap-frog stochastic dynamics integrator was used as the main run control option; we used a time step of 0.002 ps, and the coordinates were written out every 10 ps, while energy data were collected every 2 ps. The first 2 ns MD simulation for each lipid system was simulated in the NVT ensemble using Langevin thermostat while the subsequent nanoseconds in the NPT ensemble (T = 310 K, P = 1 atm) using Berendsen thermostat and semiisotropic pressure coupling. A time constant for coupling of 0.5 ps and an optimal compressibility for water of 4.5 × 10^−5^ bar^−1^ were implemented to obtain the best control on pressure. The MD trajectories were collected until the equilibration period achieved a convergence of the dimensions of the system and the steady state was reached according to Porasso et al. [63]. Computation of each MD trajectory was performed in parallel at a speed of 11 ns per day on a GALILEO IBM workstation (CINECA-HPC ISCRA project). All the MD run were carried out in triplicate to allow performance and reproducibility of analysis.

#### 3.1.3. MD Simulation Analyses

We collected all the MD trajectories [61] and mean values and standard deviations (SD) of characteristics were estimated for last 40 ns of each trajectory; the mean values and SD were averaged over the three replicated simulations unless otherwise stated. The analysis of the simulations’ trajectories was performed using GROMACS’s standard analysis tools, the VMD and CHIMERA software [64].

Membrane thickness and average Area per lipid values are reported in Table 2. In a previous work, we already tested out our MD protocol to predict experimental values (SAXS experimental values [9]). Here, we applied it to the herein described liposomial formulations. We also compared the obtained values with those reported by Melcrová et al. [65]. In fact, in our systems, Calcium cation increase the lipid bilayer order (data not reported, order parameter calculated with GROMACS 5.0, University of Groningen, Royal Institute of Technology Uppsala University, Upsala, Sweden, gmx_order), thus in agreement with reported experimental values [65].

Besides, the observed magnesium chloride effect is peculiar, since we expect an increase of the membrane thickness with respect the corresponding CaCl_2_ models. We explained such behavior according to data reported in other simulations [26,9].

### 3.2. Experimental Materials and Methods

#### 3.2.1. Materials

1,2-Dioleoyl-sn-glycero-3-phosphoethanolamine (DOPE), 1-palmitoyl-2-oleoyl-sn-glycero-3-phosphocholine (POPC), cholesterol (Chol) and cholesteryl hemisuccinate (CHEMS), used in the liposome preparation, were purchased from Avanti Polar Lipids Inc. (Alabaster, AL, USA). Tween-20, CaCl_2_ and MgCl_2_ salts and all solvents were obtained from Sigma Aldrich (St. Louis, MO, USA). Epigallocatechin 3-Gallate was purchased from Cayman Chemical Company (Ann Arbor, MI, USA). All other chemicals and buffer components were analytical grade preparations.

#### 3.2.2. Preparation of EGCG Liposomes

Multilamellar liposomes (MLVs) were obtained by Reverse Phase Evaporation (REV) [66,67,68]. Appropriate amounts of chloroform solutions of DOPE, POPC, CHEMS or Chol and methanol solution of EGCG were mixed to obtain a lipid equimolar ratio. The solvent was removed under reduced pressure at room temperature to prevent EGCG degradation [69]. The residual solvents were removed under nitrogen flow and lipids were dissolved in 3 mL of ether/methanol mixture (2:1, *v/v*). Then, 1 mL of water or, when necessary, of salt solution at different concentration (1, 3, 5, 6 mM) in phosphate-buffered saline (PBS 10 mM, pH 7.4) was added to reach different molar ratios of CaCl_2_ or MgCl_2_ salts to EGCG (1:1, 3:1, 5:1, 6:1). With the aim to obtain an initial water in oil emulsion (W/O), the resulting two-phase system is briefly sonicated (2 min) with an ultrasonic processor (Vibra Cell Mod VCx130, Sonics, Newtown, CT, USA) equipped with a tapered micro tip. The organic solvent was removed under vacuum (Rotavapor, Büchi, Flawil, Switzerland) to cause a phase inversion that gave an O/W emulsion. We obtained MLV suspensions with a final concentration of 3 mg/mL of total lipids and 1 mM of EGCG which were used for the determination of the EGCG affinity toward all system studied. For anionic liposomes with MgCl_2_/EGCG molar ratio 5:1, at the end of preparation, we added 0.28 mM of Tween-20 as stabilizer agent.

#### 3.2.3. Physicochemical Characterization of Liposomes

Size distribution (mean diameter and polydispersity index [PDI]) was measured by Dynamic Light Scattering (DLS) using a Nanosizer (Nano-ZS, Malvern, UK). An aliquot of the liposomes suspensions was diluted at a final concentration of 2.5 × 10^−2^ mM with the appropriate buffer. Measurements were performed at 25 °C with a fixed angle of 173°. Sizing measurements are the intensity-based mean diameters and the polydispersity index was directly calculated by the software of the apparatus.

#### 3.2.4. Determination of Encapsulation Efficiency

The unentrapped EGCG in the aqueous phase was separated by centrifugation from the liposomal suspension and its concentration determined by UV spectroscopy to calculate the encapsulation efficiency (EE) of EGCG liposomes. Briefly, a 0.5 mL of MLV liposomal suspension was spun at 25,000 *g* for 90 min at 4 °C; after having verified by the Stewart assay [70] (data reported in the Supporting Information) the absence of phospholipids in every supernatant, the concentration of free EGCG was determined spectrophotometrically at 280 nm using a UV-visible spectrophotometer (Multi-Mode Sinergy, HT BioTek, Winooski, VT, USA). The EGCG encapsulating efficiency (EE) was calculated as percentage according to Equation (1) [71,72]:

EE% = 100 × [the amount added − the amount unentrapped]/[the amount added](1)

## 4. Conclusions

Experimental and in silico results show a high correlation, thus confirming the efficiency of the developed combined approach. The encapsulation percentage showed specific ion effects together with influence of the lipid matrix composition. More in details, we found out that the presence of calcium ions hinders the insertion of EGCG in the liposome bilayer in both neutral and anionic systems. This can be explained considering that calcium ions interact more efficiently with lipids’ phosphate groups compared to EGCG, so the corresponding percentage of catechin encapsulation observed experimentally is very low. Analyzing the MD trajectories, we found very similar trends between the four neutral systems containing calcium chloride. In the anionic system, this phenomenon is more pronounced because the Ca^2+^ interacts more strongly with the negatively charged CHEMS molecules, thus explaining the lowest degree of encapsulation obtained with respect to the neutral vector. From the computational simulations we observed that the 5:1 molar ratio shows a slightly difference in the MD values respect to the other three anionic models. This correlates with the little increase of EGCG encapsulation measured experimentally in these liposomes. On the contrary, MgCl_2_ can notably improve the degree of encapsulation of EGCG molecules, in particular at the 5:1 molar ratio (Mg^2+^: EGCG) in both neutral and anionic models. Of particular interest is the result obtained with the anionic lipid system, where we achieved 100% EGCG encapsulation. In this condition we have 460 μg/mL of EGCG enclosed inside liposomes and although at the moment we have no data about the ability of our systems to reach therapeutic concentrations, it has been observed [73] that MCF7 cells treated with 10 μM of nanoencapsulated EGCG significantly lower cell viability by 40%, so we think that our system presents interesting properties. The high degree of encapsulation is due to the magnesium electrostatic influence that promote the membrane stretching along its major axis and EGCG insertion. Concerning the lipid matrix composition, all the data collected, suggest that the anionic vector is more efficient in promoting the catechin insertion within the liposomes. Even if EGCG tends to interact with POPC and DOPE phosphates, the presence of the negative charged steroid CHEMS increases the electrostatic interaction with the cations, thus, it indirectly promotes the catechin insertion. Thus, the combination of this anionic liposomal formulation enriched with magnesium chloride, appears particularly suitable to be optimized for drug delivery applications to avoid time-consuming separation steps of unentrapped bioactive molecules.

## Figures and Tables

**Table 1:** Formulations and encapsulation efficiency (%) of multilamellar liposomes loaded with Epigallocatechin-3-gallate (EGCG); standard deviation (SD) is also reported.

Liposomes Formulation	Molar Ratio Mg^2+^/EGCG	Encapsulation Efficiency (%) ± SD	Molar Ratio Ca^2+^/EGCG	Encapsulation Efficiency (%) ± SD
	0	53.5 ± 6.2	0	53.5 ± 6.2
	1:1	71.7 ± 7.8	1:1	32.7 ± 4.3
Neutral system	3:1	79.1 ± 5.3	3:1	23.9 ± 2.4
	5:1	82.3 ± 3.2	5:1	12.6 ± 6.4
	6:1	79.2 ± 3.5	6:1	4.1 ± 1.9
	0	65.5 ± 3.8	0	65.5 ± 3.8
	1:1	76.7 ± 5.5	1:1	9.3 ± 2.5
Anionic system	3:1	82.6 ± 2.3	3:1	5.9 ± 3.6
	5:1	98.9 ± 2.6	5:1	14.7 ± 1.7
	6:1	76.8 ± 4.2	6:1	9.9 ± 5.3

**Table 2:** Average Area per lipid (Areapl) and membrane thickeness values calculated on the last 20 ns MD trajectories frames. Salt: Epigallocatechin-3-gallate (EGCG) composition is specified for both neutral and anionic models.

**Neutral Model**	**Areapl**	**Membrane Thickness**
No divalent sals	0.83 ± 0.01 nm^2^	4.13 ± 0.43 nm
1:1 CaCl_2_: EGCG	0.83 ± 0.04 nm^2^	4.01 ± 0.42 nm
1:1 MgCl_2_: EGCG	0.81 ± 0.02 nm^2^	4.22 ± 0.39 nm
3:1 CaCl_2_: EGCG	0.79 ± 0.06 nm^2^	3.98 ± 0.41 nm
3:1 MgCl_2_: EGCG	0.84 ± 0.03 nm^2^	4.29 ± 0.44 nm
5:1 CaCl_2_: EGCG	0.81 ± 0.04 nm^2^	3.79 ± 0.42 nm
5:1 MgCl_2_: EGCG	0.87 ± 0.03 nm^2^	4.31 ± 0.38 nm
6:1 CaCl_2_: EGCG	0.81 ± 0.05 nm^2^	3.78 ± 0.44 nm
6:1 MgCl_2_: EGCG	0.82 ± 0.02 nm^2^	4.30 ± 0.37 nm
**Anionic Model**	**Areapl**	**Membrane Thickness**
No divalent sals	0.85 ± 0.03 nm^2^	4.12 ± 0.36 nm
1:1 CaCl_2_: EGCG	0.72 ± 0.02 nm^2^	3.70 ± 0.47 nm
1:1 MgCl_2_: EGCG	0.91 ± 0.02 nm^2^	4.33 ± 0.37 nm
3:1 CaCl_2_: EGCG	0.75 ± 0.05 nm^2^	3.73 ± 0.48 nm
3:1 MgCl_2_: EGCG	0.94 ± 0.03 nm^2^	4.42 ± 0.36 nm
5:1 CaCl_2_: EGCG	0.77 ± 0.03 nm^2^	3.61 ± 0.46 nm
5:1 MgCl_2_: EGCG	1.16 ± 0.04 nm^2^	4.79 ± 0.32 nm
6:1 CaCl_2_: EGCG	0.78 ± 0.05 nm^2^	3.60 ± 0.47 nm
6:1 MgCl_2_:EGCG	0.91 ± 0.03 nm^2^	4.21 ± 0.35 nm

## Supplementary Materials

Supplementary materials are available online, Figure S1: RMSD plotting of the three replicas for the 5:1 MgCl2: EGCG (A) and 5:1 CaCl2:EGCG (B) molecular models., Figure S2 Typical EGCG aggregate in water., Figure S3: Encapsulation experimental curves for all the anionic and neutral MLV vectors considered. Figure S4. Standard curve DOPE:POPC:CHOL. Figure S5. Standard curve DOPE:POPC:CHEMS. Table S1. Results of the Stewart assay applied to the eluates of every formulation after gel filtration to determine if phospholipids are present. Table S2. Influences of Tween-20 on colloidal stability of magnesium-EGCG (5:1) anionic liposomes by Dynamic Light Scattering. Table S3. EGCG OD (λ = 280 nm) before and after centrifugation in control solutions containing the polyphenol alone in aqueous suspension mixed with calcium or magnesium chloride at the same concentrations used for the liposomal formulations. Figure S6. Size distribution analysis by dynamic light scattering (DLS). Anionic liposomes with MgCl_2_/EGCG molar ratio 5:1 at initial time and after 24 h in absence (A) or presence (B) of 0.28 mM of Tween-20 as stabilizer agent. GROMACS itp parameters files for EGCG and CHEMS.
