# Supplementary material for: Liposomal Formulations for an Efficient Encapsulation of Epigallocatechin-3-Gallate: An In-Silico/Experimental Approach
Source: Molecules. 2018 Feb 16;23(2):441. doi: 10.3390/molecules23020441 (PMC6017453; doi:10.3390/molecules23020441)
Supplement: Supplementary file 1 [file molecules-23-00441-s001.docx]

SUPPLEMENTARY MATERIAL

Liposomal Formulations for an Efficient Encapsulation of Epigallocatechin-3-gallate: An in- Silico/Experimental Approach

Emiliano Laudadio ^1^, Cristina Minnelli ^1^, Adolfo Amici ^2^, Luca Massaccesi ^1^, Giovanna Mobbili ^1,^* and Roberta Galeazzi ^1,^*

^1^ Dipartimento di Scienze della Vita e dell’Ambiente (DISVA), Università Politecnica delle Marche, via Brecce Bianche, 60131 Ancona, Italy; e.laudadio@univpm.it (E.L.); c.minnelli@pm.univpm.it (C.M.); luca.massaccesi@gmail.com (L.M.)

^2^ Dipartimento Scienze Cliniche Specialistiche ed Odontostomatologiche, Università Politecnica delle Marche, via Brecce Bianche, 60131 Ancona, Italy; a.amici@univpm.it

***** Correspondence: g.mobbili@univpm.it (G.M.); r.galeazzi@univpm.it (R.G.); Tel.: +390712204707 (G.M.); Tel.: +390712204724 (R.G.)


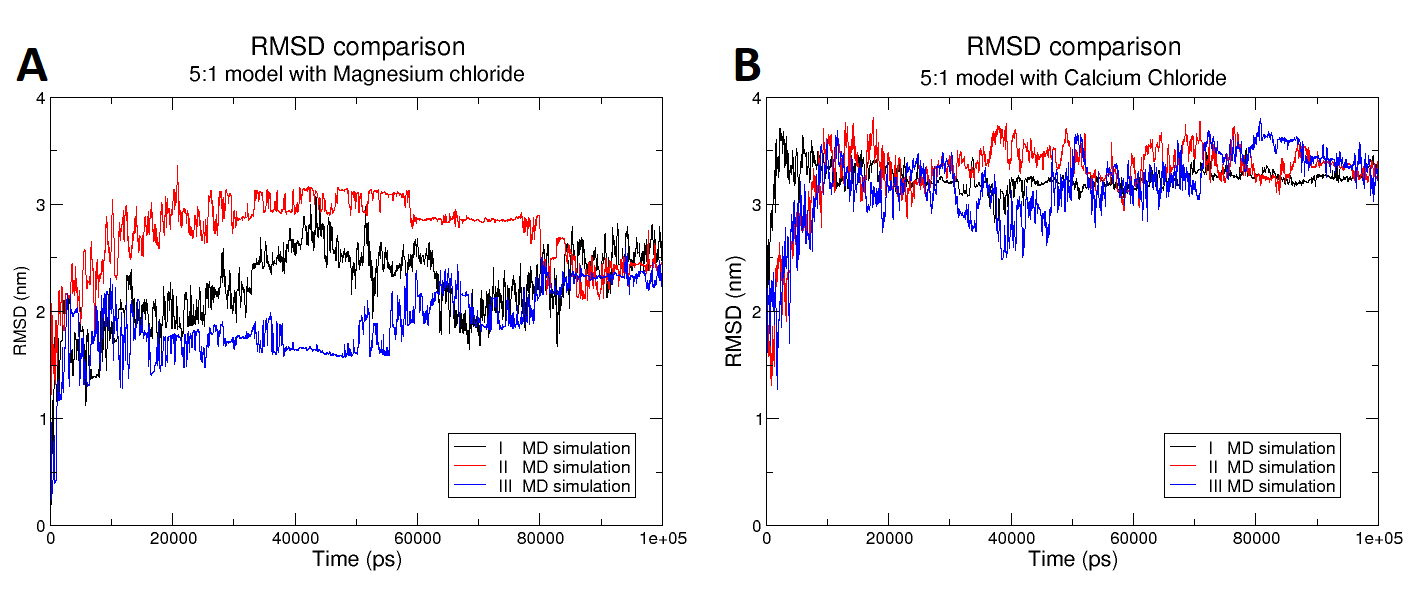


**Figure S1.** RMSD plots of the three replicas for the 5:1 MgCl_2_: EGCG (A) and 5:1 CaCl_2_:EGCG (B) molecular models.


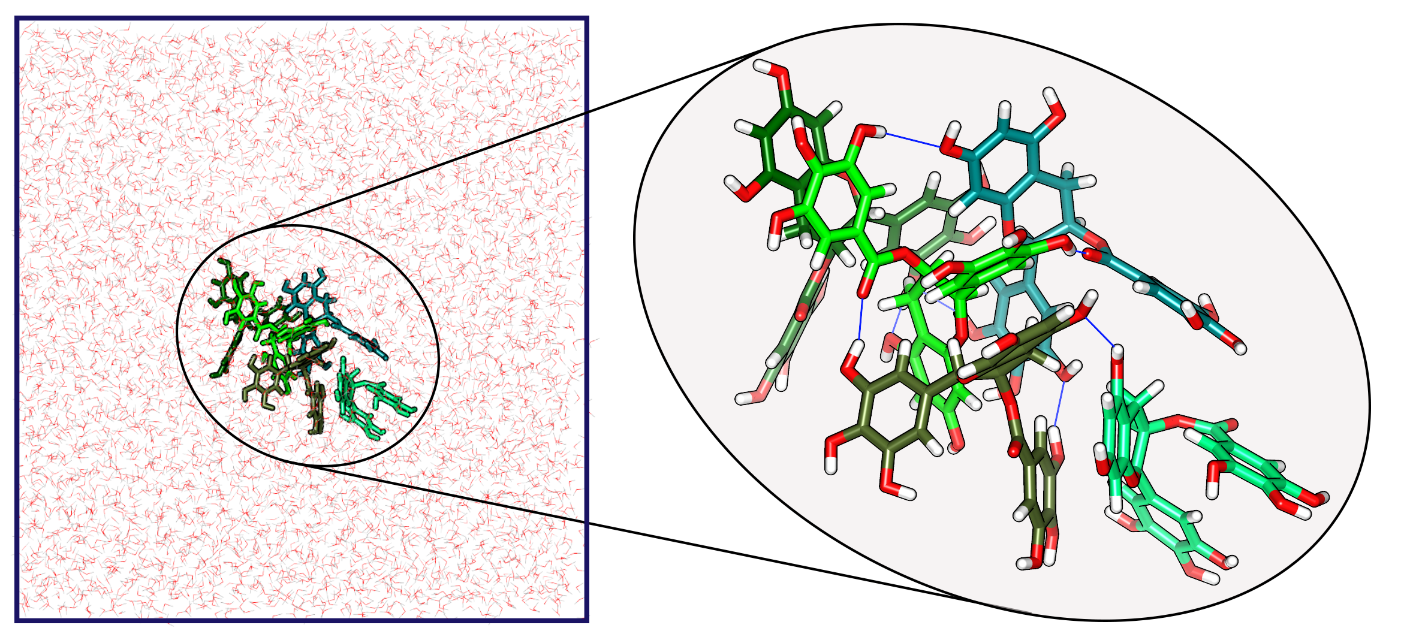


**Figure S2.** Typical EGCG aggregate in water.


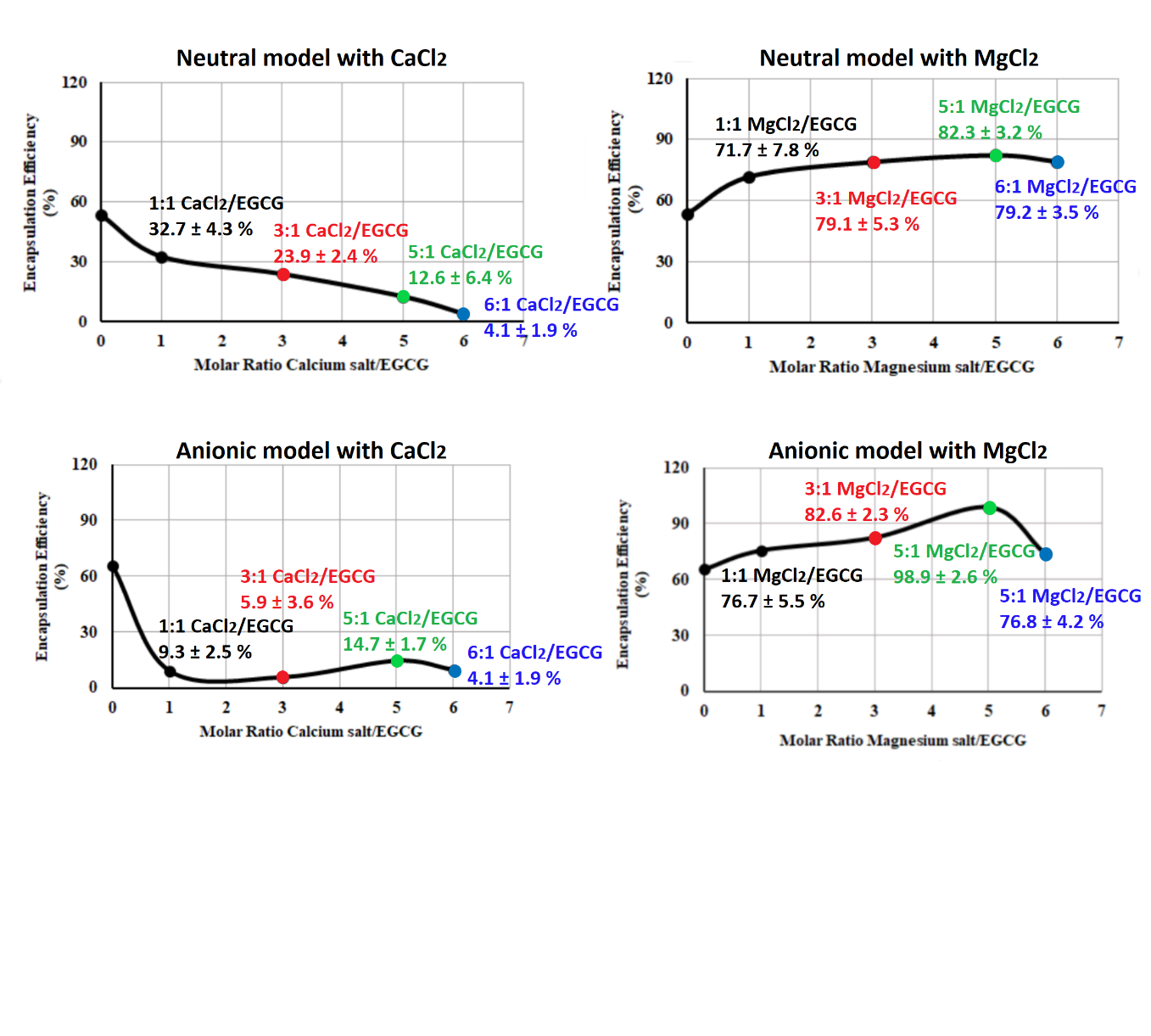


**Figure S3**. Encapsulation experimental curves for all the anionic and neutral MLV vectors considered.

**Stewart assay for the determination of the phospholipids concentration in supernatant after centrifugation**

**Figure S4.** Standard curve DOPE:POPC:CHOL

**Figure S5**. Standard curve DOPE:POPC:CHEMS

**Table S1.** Results of the Stewart assay applied to the eluates of every formulation after gel filtration to determine if phospholipids are present.

| **Formulation** | **Ratio MgCl_2_: EGCG** | **mg mL^-1^ ± SD** | **Ratio CaCl_2_: EGCG** | **mg mL^-1^ ± SD** |
| --- | --- | --- | --- | --- |
| ***Neutral System*** | 0 | 0.005 **±** 0.001 | 0 | 0.005 **±** 0.001 |
|  | 1:1 | 0.002 **±** 0.005 | 1:1 | 0.010 **±** 0.005 |
|  | 3:1 | 0.006 **±** 0.003 | 3:1 | 0.009 **±** 0.003 |
|  | 5:1 | 0.008 **±** 0.003 | 5:1 | 0.011 **±** 0.003 |
|  | 6:1 | 0.007 **±** 0.006 | 6:1 | 0.009 **±** 0.006 |
| ***Anionic System*** | 0 | 0.006 **±** 0.006 | 0 | 0.006 **±** 0.006 |
|  | 1:1 | 0.008 **±** 0.001 | 1:1 | 0.008 **±** 0.003 |
|  | 3:1 | 0.001 **±** 0.002 | 3:1 | 0.001 **±** 0.001 |
|  | 5:1 | 0.005 **±** 0.002 | 5:1 | 0.005 **±** 0.001 |
|  | 6:1 | 0.006 **±** 0.001 | 6:1 | 0.006 **±** 0.002 |

**Table S2.** Influences of Tween 20 on colloidal stability of magnesium-EGCG (5:1) anionic liposomes by Dynamic Light Scattering.

|  | **T = 0** | | **T = 24** | |
| --- | --- | --- | --- | --- |
|  | **Particle size (nm)** | **PDI (nm)** | **Particle size (nm)** | **PDI (nm)** |
| **- Tween 20** | 368 ± 43.9 | 0.507 ± 0.09 | 2.311 ± 20.1 | 1.1 ± 0.2 |
| **+ Tween 20** | 330 ± 6.5 | 0.111 ± 0.2 | 340 ± 3.2 | 0.205 ± 0.2 |


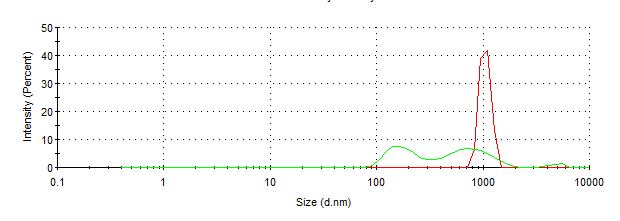


**A**

**T = 0 h**

**T = 24 h**

**
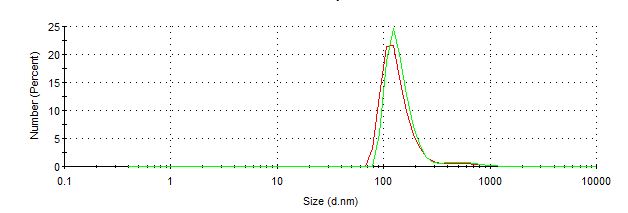
**

**B**

**T = 24 h**

**T = 0 h**

**Figure S6.** Size distribution analysis by dynamic light scattering (DLS). Anionic liposomes with MgCl_2_ / EGCG molar ratio 5:1 at initial time and after 24h in absence (**A**) or presence (**B**) of 0.28 mM of Tween-20 as stabilizer agent.

**Table S3.** EGCG OD (λ =280 nm) before and after centrifugation in control solutions containing the polyphenol alone in aqueous suspension mixed with calcium or magnesium chloride at the same concentrations used for the liposomal formulations.

|  | **OD (ʎ = 280 nm)** | |
| --- | --- | --- |
| **Molar ratio MgCl_2_ / EGCG** | **Before centrifugation** | **After centrifugation** |
| - | 0.464 ± 0.025 | 0.464 ± 0.025 |
| 1 : 1 | 0.481 ± 0.025 | 0.481 ± 0.052 |
| 3 : 1 | 0.510 ± 0.025 | 0.530 ± 0.042 |
| 5 : 1 | 0.525 ± 0.025 | 0.519 ± 0.066 |
| 6 : 1 | 0.682 ± 0.025 | 0.672 ± 0.035 |
| **Molar ratio CaCl_2_ / EGCG** | **Before centrifugation** | **After centrifugation** |
| - | 0.464 ± 0.025 | 0.464 ± 0.025 |
| 1 : 1 | 0.498 ± 0.025 | 0.502 ± 0.031 |
| 3 : 1 | 0.560 ± 0.025 | 0.582 ± 0.044 |
| 5 : 1 | 0.700 ± 0.025 | 0.761 ± 0.059 |
| 6 : 1 | 0.861 ± 0.025 | 0.845 ± 0.030 |

**EGCG CHARMM36 - GROMACS itp file**

[ moleculetype ]

; name nrexcl

EPIA 1

[ atoms ]

; nr type resnr residue atom cgnr charge mass typeB chargeB

1 OH 1 EPI O1 1 -0.50260 16.000000

2 CA 1 EPI C1 2 0.11010 12.000000

3 CA 1 EPI C2 3 0.02610 12.000000

4 OH 1 EPI O2 4 -0.51210 16.000000

5 CA 1 EPI C3 5 0.11010 12.000000

6 OH 1 EPI O3 6 -0.50260 16.000000

7 CA 1 EPI C4 7 -0.16550 12.000000

8 CA 1 EPI C5 8 -0.04830 12.000000

9 CT 1 EPI C6 9 0.19540 12.000000

10 CA 1 EPI C7 10 0.19410 12.000000

11 CA 1 EPI C8 11 -0.21600 12.000000

12 CA 1 EPI C9 12 0.18410 12.000000

13 CA 1 EPI C10 13 -0.27400 12.000000

14 CA 1 EPI C11 14 0.19810 12.000000

15 CA 1 EPI C12 15 -0.25830 12.000000

16 CT 1 EPI C13 16 0.13310 12.000000

17 C 1 EPI C14 17 0.65570 12.000000

18 CA 1 EPI C15 18 -0.10160 12.000000

19 CA 1 EPI C16 19 -0.12450 12.000000

20 CA 1 EPI C17 20 -0.12450 12.000000

21 CT 1 EPI C18 21 -0.04510 12.000000

22 OS 1 EPI O4 22 -0.30890 16.000000

23 OH 1 EPI O5 23 -0.49110 16.000000

24 OH 1 EPI O6 24 -0.49410 16.000000

25 CA 1 EPI C19 25 -0.16550 12.000000

26 OS 1 EPI O7 26 -0.42090 16.000000

27 O 1 EPI O8 27 -0.54700 16.000000

28 CA 1 EPI C20 28 0.09260 12.000000

29 OH 1 EPI O9 29 -0.50060 16.000000

30 CA 1 EPI C21 30 0.05510 12.000000

31 OH 1 EPI O10 31 -0.50810 16.000000

32 CA 1 EPI C22 32 0.09260 12.000000

33 OH 1 EPI O11 33 -0.50060 16.000000

34 HC 1 EPI H1 34 0.15450 1.000000

35 HC 1 EPI H2 35 0.06870 1.000000

36 HC 1 EPI H3 36 0.16900 1.000000

37 HC 1 EPI H4 37 0.15500 1.000000

38 HC 1 EPI H5 38 0.08570 1.000000

39 HC 1 EPI H6 39 0.17300 1.000000

40 HC 1 EPI H7 40 0.17300 1.000000

41 HC 1 EPI H8 41 0.06320 1.000000

42 HC 1 EPI H9 42 0.06320 1.000000

43 HC 1 EPI H10 43 0.15450 1.000000

44 HO 1 EPI H11 44 0.44050 1.000000

45 HO 1 EPI H12 45 0.44050 1.000000

46 HO 1 EPI H13 46 0.42400 1.000000

47 HO 1 EPI H14 47 0.45200 1.000000

48 HO 1 EPI H15 48 0.43700 1.000000

49 HO 1 EPI H16 49 0.43700 1.000000

50 HO 1 EPI H17 50 0.42600 1.000000

51 HO 1 EPI H18 51 0.44800 1.000000

[ bonds ]

; ai aj funct r k

33 44 1 9.7400e-02 3.0928e+05

31 47 1 9.7400e-02 3.0928e+05

29 45 1 9.7400e-02 3.0928e+05

25 43 1 1.0870e-01 2.8811e+05

24 50 1 9.7400e-02 3.0928e+05

23 46 1 9.7400e-02 3.0928e+05

21 41 1 1.0920e-01 2.8225e+05

21 42 1 1.0920e-01 2.8225e+05

20 40 1 1.0870e-01 2.8811e+05

19 39 1 1.0870e-01 2.8811e+05

16 38 1 1.0920e-01 2.8225e+05

13 37 1 1.0870e-01 2.8811e+05

11 36 1 1.0870e-01 2.8811e+05

9 35 1 1.0920e-01 2.8225e+05

7 34 1 1.0870e-01 2.8811e+05

6 48 1 9.7400e-02 3.0928e+05

4 51 1 9.7400e-02 3.0928e+05

1 49 1 9.7400e-02 3.0928e+05

32 33 1 1.3330e-01 3.5597e+05

30 31 1 1.3330e-01 3.5597e+05

30 32 1 1.3870e-01 4.0033e+05

28 29 1 1.3330e-01 3.5597e+05

28 30 1 1.3870e-01 4.0033e+05

20 28 1 1.3870e-01 4.0033e+05

19 32 1 1.3870e-01 4.0033e+05

18 19 1 1.3870e-01 4.0033e+05

18 20 1 1.3870e-01 4.0033e+05

17 18 1 1.4060e-01 3.7648e+05

17 26 1 1.3430e-01 3.4418e+05

17 27 1 1.2140e-01 5.4225e+05

16 21 1 1.5350e-01 2.5363e+05

16 26 1 1.4390e-01 2.5230e+05

15 21 1 1.5080e-01 2.7472e+05

14 15 1 1.3870e-01 4.0033e+05

14 24 1 1.3330e-01 3.5597e+05

13 14 1 1.3870e-01 4.0033e+05

12 13 1 1.3870e-01 4.0033e+05

12 23 1 1.3330e-01 3.5597e+05

11 12 1 1.3870e-01 4.0033e+05

10 11 1 1.3870e-01 4.0033e+05

10 15 1 1.3870e-01 4.0033e+05

10 22 1 1.3570e-01 3.2853e+05

9 16 1 1.5350e-01 2.5363e+05

9 22 1 1.4390e-01 2.5230e+05

8 9 1 1.5080e-01 2.7472e+05

8 25 1 1.3870e-01 4.0033e+05

7 8 1 1.3870e-01 4.0033e+05

5 6 1 1.3330e-01 3.5597e+05

5 7 1 1.3870e-01 4.0033e+05

3 4 1 1.3330e-01 3.5597e+05

3 5 1 1.3870e-01 4.0033e+05

2 3 1 1.3870e-01 4.0033e+05

2 25 1 1.3870e-01 4.0033e+05

1 2 1 1.3330e-01 3.5597e+05

[ pairs ]

; ai aj funct

38 41 1

38 42 1

35 38 1

33 39 1

32 47 1

30 40 1

30 45 1

30 39 1

30 44 1

29 40 1

28 47 1

26 35 1

26 41 1

26 42 1

49 25 1

25 34 1

25 35 1

24 37 1

23 36 1

23 37 1

22 38 1

22 36 1

21 35 1

20 39 1

20 45 1

19 40 1

19 44 1

17 39 1

17 40 1

17 38 1

15 36 1

15 37 1

15 50 1

15 38 1

14 41 1

14 42 1

13 36 1

13 46 1

13 50 1

11 37 1

11 46 1

10 41 1

10 42 1

10 35 1

9 34 1

9 43 1

9 41 1

9 42 1

8 38 1

7 48 1

7 35 1

7 43 1

6 34 1

5 51 1

49 3 1

3 43 1

3 48 1

3 34 1

2 51 1

1 43 1

31 33 1

29 31 1

29 32 1

28 33 1

22 25 1

22 26 1

21 22 1

21 24 1

20 26 1

20 27 1

20 32 1

20 31 1

19 26 1

19 27 1

19 28 1

19 31 1

18 30 1

18 33 1

18 29 1

17 32 1

17 28 1

17 21 1

16 25 1

16 18 1

16 27 1

15 26 1

14 23 1

14 22 1

14 16 1

13 21 1

12 15 1

12 22 1

12 24 1

11 14 1

11 21 1

10 13 1

10 23 1

10 24 1

10 16 1

9 15 1

9 17 1

9 11 1

8 21 1

8 26 1

8 10 1

7 16 1

7 22 1

6 8 1

5 25 1

5 9 1

4 25 1

4 6 1

4 7 1

3 8 1

2 6 1

2 7 1

2 9 1

1 4 1

1 5 1

1 8 1

[ angles ]

; ai aj ak funct theta cth

41 21 42 1 1.0835e+02 3.2995e+02

32 19 39 1 1.1970e+02 4.2091e+02

32 33 44 1 1.0898e+02 4.1765e+02

30 31 47 1 1.0898e+02 4.1765e+02

28 20 40 1 1.1970e+02 4.2091e+02

28 29 45 1 1.0898e+02 4.1765e+02

26 16 38 1 1.0870e+02 4.2568e+02

22 9 35 1 1.0870e+02 4.2568e+02

21 16 38 1 1.1005e+02 3.8802e+02

18 19 39 1 1.1970e+02 4.2091e+02

18 20 40 1 1.1970e+02 4.2091e+02

16 9 35 1 1.1005e+02 3.8802e+02

16 21 41 1 1.1005e+02 3.8802e+02

16 21 42 1 1.1005e+02 3.8802e+02

15 21 41 1 1.1049e+02 3.9355e+02

15 21 42 1 1.1049e+02 3.9355e+02

14 13 37 1 1.1970e+02 4.2091e+02

14 24 50 1 1.0898e+02 4.1765e+02

12 11 36 1 1.1970e+02 4.2091e+02

12 13 37 1 1.1970e+02 4.2091e+02

12 23 46 1 1.0898e+02 4.1765e+02

10 11 36 1 1.1970e+02 4.2091e+02

9 16 38 1 1.1005e+02 3.8802e+02

8 7 34 1 1.1970e+02 4.2091e+02

8 9 35 1 1.1049e+02 3.9355e+02

8 25 43 1 1.1970e+02 4.2091e+02

5 6 48 1 1.0898e+02 4.1765e+02

5 7 34 1 1.1970e+02 4.2091e+02

3 4 51 1 1.0898e+02 4.1765e+02

2 1 49 1 1.0898e+02 4.1765e+02

2 25 43 1 1.1970e+02 4.2091e+02

31 30 32 1 1.2207e+02 5.9948e+02

30 32 33 1 1.2207e+02 5.9948e+02

29 28 30 1 1.2207e+02 5.9948e+02

28 30 31 1 1.2207e+02 5.9948e+02

28 30 32 1 1.1997e+02 5.6216e+02

26 17 27 1 1.2333e+02 6.3538e+02

21 16 26 1 1.0842e+02 5.6718e+02

20 28 29 1 1.2207e+02 5.9948e+02

20 28 30 1 1.1997e+02 5.6216e+02

19 18 20 1 1.1997e+02 5.6216e+02

19 32 30 1 1.1997e+02 5.6216e+02

19 32 33 1 1.2207e+02 5.9948e+02

18 17 26 1 1.1408e+02 5.9880e+02

18 17 27 1 1.1912e+02 6.0894e+02

18 19 32 1 1.1997e+02 5.6216e+02

18 20 28 1 1.1997e+02 5.6216e+02

17 18 19 1 1.2070e+02 5.6844e+02

17 18 20 1 1.2070e+02 5.6844e+02

16 9 22 1 1.0842e+02 5.6718e+02

16 26 17 1 1.1514e+02 5.3246e+02

15 10 22 1 1.2189e+02 5.9446e+02

15 14 24 1 1.2207e+02 5.9948e+02

15 21 16 1 1.1144e+02 5.3162e+02

14 15 21 1 1.2342e+02 5.3831e+02

13 12 23 1 1.2207e+02 5.9948e+02

13 14 15 1 1.1997e+02 5.6216e+02

13 14 24 1 1.2207e+02 5.9948e+02

12 13 14 1 1.1997e+02 5.6216e+02

11 10 15 1 1.1997e+02 5.6216e+02

11 10 22 1 1.2189e+02 5.9446e+02

11 12 13 1 1.1997e+02 5.6216e+02

11 12 23 1 1.2207e+02 5.9948e+02

10 11 12 1 1.1997e+02 5.6216e+02

10 15 14 1 1.1997e+02 5.6216e+02

10 15 21 1 1.2342e+02 5.3831e+02

9 8 25 1 1.2342e+02 5.3831e+02

9 16 21 1 1.1063e+02 5.2894e+02

9 16 26 1 1.0842e+02 5.6718e+02

9 22 10 1 1.1209e+02 5.3731e+02

8 9 16 1 1.1144e+02 5.3162e+02

8 9 22 1 1.0848e+02 5.7279e+02

7 8 9 1 1.2342e+02 5.3831e+02

7 8 25 1 1.1997e+02 5.6216e+02

6 5 7 1 1.2207e+02 5.9948e+02

5 7 8 1 1.1997e+02 5.6216e+02

4 3 5 1 1.2207e+02 5.9948e+02

3 2 25 1 1.1997e+02 5.6216e+02

3 5 6 1 1.2207e+02 5.9948e+02

3 5 7 1 1.1997e+02 5.6216e+02

2 3 4 1 1.2207e+02 5.9948e+02

2 3 5 1 1.1997e+02 5.6216e+02

2 25 8 1 1.1997e+02 5.6216e+02

1 2 3 1 1.2207e+02 5.9948e+02

1 2 25 1 1.2207e+02 5.9948e+02

[ dihedrals ]

;i j k l func C0 ... C5

38 16 21 41 3 0.62760 1.88280 0.00000 -2.51040 0.00000 0.00000 ;

38 16 21 42 3 0.62760 1.88280 0.00000 -2.51040 0.00000 0.00000 ;

35 9 16 38 3 0.62760 1.88280 0.00000 -2.51040 0.00000 0.00000 ;

33 32 19 39 3 55.64720 0.00000 -55.64720 0.00000 0.00000 0.00000 ;

32 30 31 47 3 8.78640 0.00000 -8.78640 0.00000 0.00000 0.00000 ;

30 28 20 40 3 55.64720 0.00000 -55.64720 0.00000 0.00000 0.00000 ;

30 28 29 45 3 8.78640 0.00000 -8.78640 0.00000 0.00000 0.00000 ;

30 32 19 39 3 55.64720 0.00000 -55.64720 0.00000 0.00000 0.00000 ;

30 32 33 44 3 8.78640 0.00000 -8.78640 0.00000 0.00000 0.00000 ;

29 28 20 40 3 55.64720 0.00000 -55.64720 0.00000 0.00000 0.00000 ;

28 30 31 47 3 8.78640 0.00000 -8.78640 0.00000 0.00000 0.00000 ;

26 16 9 35 3 1.04600 -1.04600 0.00000 0.00000 0.00000 0.00000 ;

26 16 21 41 3 1.04600 -1.04600 0.00000 0.00000 0.00000 0.00000 ;

26 16 21 42 3 1.04600 -1.04600 0.00000 0.00000 0.00000 0.00000 ;

49 1 2 25 3 8.78640 0.00000 -8.78640 0.00000 0.00000 0.00000 ;

25 8 7 34 3 55.64720 0.00000 -55.64720 0.00000 0.00000 0.00000 ;

25 8 9 35 3 6.40152 -9.58136 0.00000 6.35968 0.00000 0.00000 ;

24 14 13 37 3 55.64720 0.00000 -55.64720 0.00000 0.00000 0.00000 ;

23 12 11 36 3 55.64720 0.00000 -55.64720 0.00000 0.00000 0.00000 ;

23 12 13 37 3 55.64720 0.00000 -55.64720 0.00000 0.00000 0.00000 ;

22 9 16 38 3 1.04600 -1.04600 0.00000 0.00000 0.00000 0.00000 ;

22 10 11 36 3 55.64720 0.00000 -55.64720 0.00000 0.00000 0.00000 ;

21 16 9 35 3 0.66944 2.00832 0.00000 -2.67776 0.00000 0.00000 ;

20 18 19 39 3 55.64720 0.00000 -55.64720 0.00000 0.00000 0.00000 ;

20 28 29 45 3 8.78640 0.00000 -8.78640 0.00000 0.00000 0.00000 ;

19 18 20 40 3 55.64720 0.00000 -55.64720 0.00000 0.00000 0.00000 ;

19 32 33 44 3 8.78640 0.00000 -8.78640 0.00000 0.00000 0.00000 ;

17 18 19 39 3 55.64720 0.00000 -55.64720 0.00000 0.00000 0.00000 ;

17 18 20 40 3 55.64720 0.00000 -55.64720 0.00000 0.00000 0.00000 ;

17 26 16 38 3 1.60247 4.80742 0.00000 -6.40989 0.00000 0.00000 ;

15 10 11 36 3 55.64720 0.00000 -55.64720 0.00000 0.00000 0.00000 ;

15 14 13 37 3 55.64720 0.00000 -55.64720 0.00000 0.00000 0.00000 ;

15 14 24 50 3 8.78640 0.00000 -8.78640 0.00000 0.00000 0.00000 ;

15 21 16 38 3 0.65270 1.95811 0.00000 -2.61082 0.00000 0.00000 ;

14 15 21 41 3 6.40152 -9.58136 0.00000 6.35968 0.00000 0.00000 ;

14 15 21 42 3 6.40152 -9.58136 0.00000 6.35968 0.00000 0.00000 ;

13 12 11 36 3 55.64720 0.00000 -55.64720 0.00000 0.00000 0.00000 ;

13 12 23 46 3 8.78640 0.00000 -8.78640 0.00000 0.00000 0.00000 ;

13 14 24 50 3 8.78640 0.00000 -8.78640 0.00000 0.00000 0.00000 ;

11 12 13 37 3 55.64720 0.00000 -55.64720 0.00000 0.00000 0.00000 ;

11 12 23 46 3 8.78640 0.00000 -8.78640 0.00000 0.00000 0.00000 ;

10 15 21 41 3 6.40152 -9.58136 0.00000 6.35968 0.00000 0.00000 ;

10 15 21 42 3 6.40152 -9.58136 0.00000 6.35968 0.00000 0.00000 ;

10 22 9 35 3 1.60247 4.80742 0.00000 -6.40989 0.00000 0.00000 ;

9 8 7 34 3 55.64720 0.00000 -55.64720 0.00000 0.00000 0.00000 ;

9 8 25 43 3 55.64720 0.00000 -55.64720 0.00000 0.00000 0.00000 ;

9 16 21 41 3 0.66944 2.00832 0.00000 -2.67776 0.00000 0.00000 ;

9 16 21 42 3 0.66944 2.00832 0.00000 -2.67776 0.00000 0.00000 ;

8 9 16 38 3 0.65270 1.95811 0.00000 -2.61082 0.00000 0.00000 ;

7 5 6 48 3 8.78640 0.00000 -8.78640 0.00000 0.00000 0.00000 ;

7 8 9 35 3 6.40152 -9.58136 0.00000 6.35968 0.00000 0.00000 ;

7 8 25 43 3 55.64720 0.00000 -55.64720 0.00000 0.00000 0.00000 ;

6 5 7 34 3 55.64720 0.00000 -55.64720 0.00000 0.00000 0.00000 ;

5 3 4 51 3 8.78640 0.00000 -8.78640 0.00000 0.00000 0.00000 ;

49 1 2 3 3 8.78640 0.00000 -8.78640 0.00000 0.00000 0.00000 ;

3 2 25 43 3 55.64720 0.00000 -55.64720 0.00000 0.00000 0.00000 ;

3 5 6 48 3 8.78640 0.00000 -8.78640 0.00000 0.00000 0.00000 ;

3 5 7 34 3 55.64720 0.00000 -55.64720 0.00000 0.00000 0.00000 ;

2 3 4 51 3 8.78640 0.00000 -8.78640 0.00000 0.00000 0.00000 ;

1 2 25 43 3 55.64720 0.00000 -55.64720 0.00000 0.00000 0.00000 ;

2 8 25 43 3 9.20480 0.00000 -9.20480 0.00000 0.00000 0.00000 ;

18 28 20 40 3 9.20480 0.00000 -9.20480 0.00000 0.00000 0.00000 ;

18 32 19 39 3 9.20480 0.00000 -9.20480 0.00000 0.00000 0.00000 ;

12 14 13 37 3 9.20480 0.00000 -9.20480 0.00000 0.00000 0.00000 ;

10 12 11 36 3 9.20480 0.00000 -9.20480 0.00000 0.00000 0.00000 ;

5 8 7 34 3 9.20480 0.00000 -9.20480 0.00000 0.00000 0.00000 ;

31 30 32 33 3 55.64720 0.00000 -55.64720 0.00000 0.00000 0.00000 ;

29 28 30 31 3 55.64720 0.00000 -55.64720 0.00000 0.00000 0.00000 ;

29 28 30 32 3 55.64720 0.00000 -55.64720 0.00000 0.00000 0.00000 ;

28 30 32 33 3 55.64720 0.00000 -55.64720 0.00000 0.00000 0.00000 ;

22 9 8 25 3 0.00000 0.00000 0.00000 0.00000 0.00000 0.00000 ;

22 9 16 26 3 0.60250 1.80749 9.83240 -2.40998 0.00000 0.00000 ;

21 15 10 22 3 55.64720 0.00000 -55.64720 0.00000 0.00000 0.00000 ;

21 15 14 24 3 55.64720 0.00000 -55.64720 0.00000 0.00000 0.00000 ;

21 16 9 22 3 0.65270 1.95811 0.00000 -2.61082 0.00000 0.00000 ;

20 18 17 26 3 18.20040 0.00000 -18.20040 0.00000 0.00000 0.00000 ;

20 18 17 27 3 19.45560 3.76560 -18.20040 -5.02080 0.00000 0.00000 ;

20 18 19 32 3 30.33400 0.00000 -30.33400 0.00000 0.00000 0.00000 ;

20 28 30 31 3 55.64720 0.00000 -55.64720 0.00000 0.00000 0.00000 ;

20 28 30 32 3 30.33400 0.00000 -30.33400 0.00000 0.00000 0.00000 ;

19 18 17 26 3 18.20040 0.00000 -18.20040 0.00000 0.00000 0.00000 ;

19 18 17 27 3 19.45560 3.76560 -18.20040 -5.02080 0.00000 0.00000 ;

19 18 20 28 3 30.33400 0.00000 -30.33400 0.00000 0.00000 0.00000 ;

19 32 30 28 3 30.33400 0.00000 -30.33400 0.00000 0.00000 0.00000 ;

19 32 30 31 3 55.64720 0.00000 -55.64720 0.00000 0.00000 0.00000 ;

18 19 32 30 3 30.33400 0.00000 -30.33400 0.00000 0.00000 0.00000 ;

18 19 32 33 3 55.64720 0.00000 -55.64720 0.00000 0.00000 0.00000 ;

18 20 28 29 3 55.64720 0.00000 -55.64720 0.00000 0.00000 0.00000 ;

18 20 28 30 3 30.33400 0.00000 -30.33400 0.00000 0.00000 0.00000 ;

17 18 19 32 3 55.64720 0.00000 -55.64720 0.00000 0.00000 0.00000 ;

17 18 20 28 3 55.64720 0.00000 -55.64720 0.00000 0.00000 0.00000 ;

17 26 16 21 3 4.94967 8.15462 0.00000 -6.40989 0.00000 0.00000 ;

16 9 8 25 3 0.00000 0.00000 0.00000 0.00000 0.00000 0.00000 ;

16 26 17 18 3 22.59360 0.00000 -22.59360 0.00000 0.00000 0.00000 ;

16 26 17 27 3 28.45120 5.85760 -22.59360 0.00000 0.00000 0.00000 ;

15 21 16 26 3 0.65270 1.95811 0.00000 -2.61082 0.00000 0.00000 ;

14 13 12 23 3 55.64720 0.00000 -55.64720 0.00000 0.00000 0.00000 ;

14 15 10 22 3 55.64720 0.00000 -55.64720 0.00000 0.00000 0.00000 ;

14 15 21 16 3 0.00000 0.00000 0.00000 0.00000 0.00000 0.00000 ;

13 14 15 21 3 55.64720 0.00000 -55.64720 0.00000 0.00000 0.00000 ;

12 11 10 15 3 30.33400 0.00000 -30.33400 0.00000 0.00000 0.00000 ;

12 11 10 22 3 55.64720 0.00000 -55.64720 0.00000 0.00000 0.00000 ;

12 13 14 15 3 30.33400 0.00000 -30.33400 0.00000 0.00000 0.00000 ;

12 13 14 24 3 55.64720 0.00000 -55.64720 0.00000 0.00000 0.00000 ;

11 10 15 14 3 30.33400 0.00000 -30.33400 0.00000 0.00000 0.00000 ;

11 10 15 21 3 55.64720 0.00000 -55.64720 0.00000 0.00000 0.00000 ;

11 12 13 14 3 30.33400 0.00000 -30.33400 0.00000 0.00000 0.00000 ;

10 11 12 13 3 30.33400 0.00000 -30.33400 0.00000 0.00000 0.00000 ;

10 11 12 23 3 55.64720 0.00000 -55.64720 0.00000 0.00000 0.00000 ;

10 15 14 13 3 30.33400 0.00000 -30.33400 0.00000 0.00000 0.00000 ;

10 15 14 24 3 55.64720 0.00000 -55.64720 0.00000 0.00000 0.00000 ;

10 15 21 16 3 0.00000 0.00000 0.00000 0.00000 0.00000 0.00000 ;

10 22 9 16 3 1.60247 4.80742 0.00000 -6.40989 0.00000 0.00000 ;

9 16 21 15 3 0.65270 1.95811 0.00000 -2.61082 0.00000 0.00000 ;

9 16 26 17 3 4.94967 8.15462 0.00000 -6.40989 0.00000 0.00000 ;

9 22 10 11 3 8.78640 0.00000 -8.78640 0.00000 0.00000 0.00000 ;

9 22 10 15 3 8.78640 0.00000 -8.78640 0.00000 0.00000 0.00000 ;

8 9 16 21 3 0.65270 1.95811 0.00000 -2.61082 0.00000 0.00000 ;

8 9 16 26 3 0.65270 1.95811 0.00000 -2.61082 0.00000 0.00000 ;

8 9 22 10 3 1.60247 4.80742 0.00000 -6.40989 0.00000 0.00000 ;

7 8 9 16 3 0.00000 0.00000 0.00000 0.00000 0.00000 0.00000 ;

7 8 9 22 3 0.00000 0.00000 0.00000 0.00000 0.00000 0.00000 ;

6 5 7 8 3 55.64720 0.00000 -55.64720 0.00000 0.00000 0.00000 ;

5 3 2 25 3 30.33400 0.00000 -30.33400 0.00000 0.00000 0.00000 ;

5 7 8 9 3 55.64720 0.00000 -55.64720 0.00000 0.00000 0.00000 ;

5 7 8 25 3 30.33400 0.00000 -30.33400 0.00000 0.00000 0.00000 ;

4 3 2 25 3 55.64720 0.00000 -55.64720 0.00000 0.00000 0.00000 ;

4 3 5 6 3 55.64720 0.00000 -55.64720 0.00000 0.00000 0.00000 ;

4 3 5 7 3 55.64720 0.00000 -55.64720 0.00000 0.00000 0.00000 ;

3 2 25 8 3 30.33400 0.00000 -30.33400 0.00000 0.00000 0.00000 ;

3 5 7 8 3 30.33400 0.00000 -30.33400 0.00000 0.00000 0.00000 ;

2 3 5 6 3 55.64720 0.00000 -55.64720 0.00000 0.00000 0.00000 ;

2 3 5 7 3 30.33400 0.00000 -30.33400 0.00000 0.00000 0.00000 ;

2 25 8 7 3 30.33400 0.00000 -30.33400 0.00000 0.00000 0.00000 ;

2 25 8 9 3 55.64720 0.00000 -55.64720 0.00000 0.00000 0.00000 ;

1 2 3 4 3 55.64720 0.00000 -55.64720 0.00000 0.00000 0.00000 ;

1 2 3 5 3 55.64720 0.00000 -55.64720 0.00000 0.00000 0.00000 ;

1 2 25 8 3 55.64720 0.00000 -55.64720 0.00000 0.00000 0.00000 ;

19 30 32 33 3 9.20480 0.00000 -9.20480 0.00000 0.00000 0.00000 ;

28 32 30 31 3 9.20480 0.00000 -9.20480 0.00000 0.00000 0.00000 ;

20 30 28 29 3 9.20480 0.00000 -9.20480 0.00000 0.00000 0.00000 ;

17 19 18 20 3 9.20480 0.00000 -9.20480 0.00000 0.00000 0.00000 ;

18 27 17 26 3 9.20480 0.00000 -9.20480 0.00000 0.00000 0.00000 ;

10 14 15 21 3 9.20480 0.00000 -9.20480 0.00000 0.00000 0.00000 ;

13 15 14 24 3 9.20480 0.00000 -9.20480 0.00000 0.00000 0.00000 ;

11 13 12 23 3 9.20480 0.00000 -9.20480 0.00000 0.00000 0.00000 ;

11 15 10 22 3 9.20480 0.00000 -9.20480 0.00000 0.00000 0.00000 ;

7 25 8 9 3 9.20480 0.00000 -9.20480 0.00000 0.00000 0.00000 ;

3 7 5 6 3 9.20480 0.00000 -9.20480 0.00000 0.00000 0.00000 ;

2 5 3 4 3 9.20480 0.00000 -9.20480 0.00000 0.00000 0.00000 ;

1 2 25 3 3 9.20480 0.00000 -9.20480 0.00000 0.00000 0.00000 ;

**CHEMS** **CHARMM36 - GROMACS itp file**

[ moleculetype ]

; name nrexcl

CHEA 3

[ atoms ]

; nr type resnr residue atom cgnr charge mass typeB chargeB

1 CT 1 CHE C3 1 0.15710 12.000000

2 OS 1 CHE O3 2 -0.45390 16.000000

3 CT 1 CHE C4 3 -0.04720 12.000000

4 CA 1 CHE C5 4 -0.08940 12.000000

5 CA 1 CHE C6 5 -0.18720 12.000000

6 CT 1 CHE C7 6 -0.03720 12.000000

7 CT 1 CHE C8 7 -0.04770 12.000000

8 CT 1 CHE C14 8 -0.06370 12.000000

9 CT 1 CHE C15 9 -0.07440 12.000000

10 CT 1 CHE C16 10 -0.07840 12.000000

11 CT 1 CHE C17 11 -0.05570 12.000000

12 CT 1 CHE C13 12 -0.04500 12.000000

13 CT 1 CHE C18 13 -0.08210 12.000000

14 CT 1 CHE C12 14 -0.06440 12.000000

15 CT 1 CHE C11 15 -0.07640 12.000000

16 CT 1 CHE C9 16 -0.04870 12.000000

17 CT 1 CHE C10 17 -0.00380 12.000000

18 CT 1 CHE C19 18 -0.08310 12.000000

19 CT 1 CHE C1 19 -0.06440 12.000000

20 CT 1 CHE C2 20 -0.10540 12.000000

21 CT 1 CHE C20 21 -0.05470 12.000000

22 CT 1 CHE C21 22 -0.09110 12.000000

23 CT 1 CHE C22 23 -0.08040 12.000000

24 CT 1 CHE C23 24 -0.07940 12.000000

25 CT 1 CHE C24 25 -0.07540 12.000000

26 CT 1 CHE C25 26 -0.06770 12.000000

27 CT 1 CHE C26 27 -0.08960 12.000000

28 CT 1 CHE C27 28 -0.08960 12.000000

29 HC 1 CHE H3 29 0.06470 1.000000

30 HC 1 CHE H4A 30 0.05920 1.000000

31 HC 1 CHE H4B 31 0.05920 1.000000

32 HC 1 CHE H6 32 0.12000 1.000000

33 HC 1 CHE H7A 33 0.04220 1.000000

34 HC 1 CHE H7B 34 0.04220 1.000000

35 HC 1 CHE H8 35 0.05770 1.000000

36 HC 1 CHE H14 36 0.04570 1.000000

37 HC 1 CHE H15A 37 0.04120 1.000000

38 HC 1 CHE H15B 38 0.04120 1.000000

39 HC 1 CHE H16A 39 0.03770 1.000000

40 HC 1 CHE H16B 40 0.03770 1.000000

41 HC 1 CHE H17 41 0.04570 1.000000

42 HC 1 CHE H18A 42 0.03500 1.000000

43 HC 1 CHE H18B 43 0.03500 1.000000

44 HC 1 CHE H18C 44 0.03500 1.000000

45 HC 1 CHE H12A 45 0.03820 1.000000

46 HC 1 CHE H12B 46 0.03820 1.000000

47 HC 1 CHE H11A 47 0.04270 1.000000

48 HC 1 CHE H11B 48 0.04270 1.000000

49 HC 1 CHE H9 49 0.04870 1.000000

50 HC 1 CHE H19A 50 0.03700 1.000000

51 HC 1 CHE H19B 51 0.03700 1.000000

52 HC 1 CHE H19C 52 0.03700 1.000000

53 HC 1 CHE H1A 53 0.04020 1.000000

54 HC 1 CHE H1B 54 0.04020 1.000000

55 HC 1 CHE H2A 55 0.06170 1.000000

56 HC 1 CHE H2B 56 0.06170 1.000000

57 HC 1 CHE H20 57 0.04970 1.000000

58 HC 1 CHE H21A 58 0.03400 1.000000

59 HC 1 CHE H21B 59 0.03400 1.000000

60 HC 1 CHE H21C 60 0.03400 1.000000

61 HC 1 CHE H22A 61 0.04020 1.000000

62 HC 1 CHE H22B 62 0.04020 1.000000

63 HC 1 CHE H23A 63 0.03970 1.000000

64 HC 1 CHE H23B 64 0.03970 1.000000

65 HC 1 CHE H24A 65 0.03920 1.000000

66 HC 1 CHE H24B 66 0.03920 1.000000

67 HC 1 CHE H25 67 0.04570 1.000000

68 HC 1 CHE H26A 68 0.03220 1.000000

69 HC 1 CHE H26B 69 0.03220 1.000000

70 HC 1 CHE H26C 70 0.03220 1.000000

71 HC 1 CHE H27A 71 0.03220 1.000000

72 HC 1 CHE H27B 72 0.03220 1.000000

73 HC 1 CHE H27C 73 0.03220 1.000000

74 C 1 CHE C28 74 0.63910 12.000000

75 O 1 CHE O1 75 -0.57900 16.000000

76 CT 1 CHE C29 76 -0.10540 12.000000

77 CT 1 CHE C30 77 -0.20040 12.000000

78 O 1 CHE O2 78 -0.84280 16.000000

79 C 1 CHE C31 79 0.91590 12.000000

80 OH 1 CHE O4 80 -0.84280 16.000000

81 HC 1 CHE H28 81 0.08520 1.000000

82 HC 1 CHE H29 82 0.08520 1.000000

83 HC 1 CHE H30 83 0.03520 1.000000

84 HC 1 CHE H31 84 0.03520 1.000000

[ bonds ]

; ai aj funct r k

77 83 1 1.0920e-01 2.8225e+05

77 84 1 1.0920e-01 2.8225e+05

76 81 1 1.0920e-01 2.8225e+05

76 82 1 1.0920e-01 2.8225e+05

28 71 1 1.0920e-01 2.8225e+05

28 72 1 1.0920e-01 2.8225e+05

28 73 1 1.0920e-01 2.8225e+05

27 68 1 1.0920e-01 2.8225e+05

27 69 1 1.0920e-01 2.8225e+05

27 70 1 1.0920e-01 2.8225e+05

26 67 1 1.0920e-01 2.8225e+05

25 66 1 1.0920e-01 2.8225e+05

25 65 1 1.0920e-01 2.8225e+05

24 63 1 1.0920e-01 2.8225e+05

24 64 1 1.0920e-01 2.8225e+05

23 61 1 1.0920e-01 2.8225e+05

23 62 1 1.0920e-01 2.8225e+05

22 58 1 1.0920e-01 2.8225e+05

22 59 1 1.0920e-01 2.8225e+05

22 60 1 1.0920e-01 2.8225e+05

21 57 1 1.0920e-01 2.8225e+05

20 55 1 1.0920e-01 2.8225e+05

20 56 1 1.0920e-01 2.8225e+05

19 53 1 1.0920e-01 2.8225e+05

19 54 1 1.0920e-01 2.8225e+05

18 50 1 1.0920e-01 2.8225e+05

18 51 1 1.0920e-01 2.8225e+05

18 52 1 1.0920e-01 2.8225e+05

16 49 1 1.0920e-01 2.8225e+05

15 47 1 1.0920e-01 2.8225e+05

15 48 1 1.0920e-01 2.8225e+05

14 45 1 1.0920e-01 2.8225e+05

14 46 1 1.0920e-01 2.8225e+05

13 42 1 1.0920e-01 2.8225e+05

13 43 1 1.0920e-01 2.8225e+05

13 44 1 1.0920e-01 2.8225e+05

11 41 1 1.0920e-01 2.8225e+05

10 39 1 1.0920e-01 2.8225e+05

10 40 1 1.0920e-01 2.8225e+05

9 37 1 1.0920e-01 2.8225e+05

9 38 1 1.0920e-01 2.8225e+05

8 36 1 1.0920e-01 2.8225e+05

7 35 1 1.0920e-01 2.8225e+05

6 34 1 1.0920e-01 2.8225e+05

6 33 1 1.0920e-01 2.8225e+05

5 32 1 1.0870e-01 2.8811e+05

3 30 1 1.0920e-01 2.8225e+05

3 31 1 1.0920e-01 2.8225e+05

1 29 1 1.0920e-01 2.8225e+05

79 80 1 1.3060e-01 3.9028e+05

78 79 1 1.2140e-01 5.4225e+05

77 79 1 1.5080e-01 2.7472e+05

76 77 1 1.5350e-01 2.5363e+05

74 75 1 1.2140e-01 5.4225e+05

74 76 1 1.5080e-01 2.7472e+05

26 27 1 1.5350e-01 2.5363e+05

26 28 1 1.5350e-01 2.5363e+05

25 26 1 1.5350e-01 2.5363e+05

24 25 1 1.5350e-01 2.5363e+05

23 24 1 1.5350e-01 2.5363e+05

21 22 1 1.5350e-01 2.5363e+05

21 23 1 1.5350e-01 2.5363e+05

19 20 1 1.5350e-01 2.5363e+05

17 19 1 1.5350e-01 2.5363e+05

17 18 1 1.5350e-01 2.5363e+05

16 17 1 1.5350e-01 2.5363e+05

15 16 1 1.5350e-01 2.5363e+05

14 15 1 1.5350e-01 2.5363e+05

12 14 1 1.5350e-01 2.5363e+05

12 13 1 1.5350e-01 2.5363e+05

11 12 1 1.5350e-01 2.5363e+05

11 21 1 1.5350e-01 2.5363e+05

10 11 1 1.5350e-01 2.5363e+05

9 10 1 1.5350e-01 2.5363e+05

8 12 1 1.5350e-01 2.5363e+05

8 9 1 1.5350e-01 2.5363e+05

7 8 1 1.5350e-01 2.5363e+05

7 16 1 1.5350e-01 2.5363e+05

6 7 1 1.5350e-01 2.5363e+05

5 6 1 1.5080e-01 2.7472e+05

4 17 1 1.5080e-01 2.7472e+05

4 5 1 1.3870e-01 4.0033e+05

3 4 1 1.5080e-01 2.7472e+05

2 74 1 1.3430e-01 3.4418e+05

1 20 1 1.5350e-01 2.5363e+05

1 2 1 1.4390e-01 2.5230e+05

1 3 1 1.5350e-01 2.5363e+05

[ pairs ]

; ai aj funct

82 83 1

82 84 1

81 83 1

81 84 1

80 83 1

80 84 1

79 81 1

79 82 1

78 83 1

78 84 1

75 81 1

75 82 1

74 83 1

74 84 1

67 68 1

67 69 1

67 70 1

67 71 1

67 72 1

67 73 1

66 67 1

65 67 1

64 66 1

64 65 1

63 66 1

63 65 1

62 63 1

62 64 1

61 63 1

61 64 1

57 58 1

57 59 1

57 60 1

57 61 1

57 62 1

54 55 1

54 56 1

53 55 1

53 56 1

48 49 1

47 49 1

46 47 1

46 48 1

45 47 1

45 48 1

41 57 1

40 41 1

39 41 1

38 39 1

38 40 1

37 39 1

37 40 1

36 37 1

36 38 1

35 36 1

35 49 1

34 35 1

33 35 1

32 34 1

32 33 1

29 55 1

29 56 1

29 74 1

29 30 1

29 31 1

28 66 1

28 65 1

28 68 1

28 69 1

28 70 1

27 66 1

27 65 1

27 71 1

27 72 1

27 73 1

26 63 1

26 64 1

25 61 1

25 62 1

25 68 1

25 69 1

25 70 1

25 71 1

25 72 1

25 73 1

24 57 1

24 67 1

23 41 1

23 58 1

23 59 1

23 60 1

23 66 1

23 65 1

22 41 1

22 61 1

22 62 1

21 39 1

21 40 1

21 63 1

21 64 1

20 30 1

20 31 1

19 49 1

19 50 1

19 51 1

19 52 1

29 19 1

18 49 1

18 53 1

18 54 1

17 30 1

17 31 1

17 32 1

17 35 1

17 47 1

17 48 1

17 55 1

17 56 1

16 34 1

16 33 1

16 36 1

16 45 1

16 46 1

16 53 1

16 54 1

16 50 1

16 51 1

16 52 1

15 35 1

14 36 1

14 41 1

14 42 1

14 43 1

14 44 1

14 49 1

13 36 1

13 41 1

13 45 1

13 46 1

12 35 1

12 37 1

12 38 1

12 39 1

12 40 1

12 57 1

12 47 1

12 48 1

11 37 1

11 38 1

11 36 1

11 45 1

11 46 1

11 42 1

11 43 1

11 44 1

11 58 1

11 59 1

11 60 1

11 61 1

11 62 1

10 36 1

10 57 1

9 35 1

9 41 1

8 34 1

8 33 1

8 49 1

8 41 1

8 45 1

8 46 1

8 42 1

8 43 1

8 44 1

8 39 1

8 40 1

7 32 1

7 37 1

7 38 1

7 47 1

7 48 1

6 36 1

6 49 1

5 30 1

5 31 1

5 35 1

29 4 1

4 49 1

4 53 1

4 54 1

4 50 1

4 51 1

4 52 1

4 34 1

4 33 1

3 55 1

3 56 1

3 32 1

2 55 1

2 56 1

2 30 1

2 31 1

2 81 1

2 82 1

1 53 1

1 54 1

76 78 1

76 80 1

75 77 1

74 79 1

24 27 1

24 28 1

23 26 1

22 24 1

21 25 1

20 74 1

18 20 1

16 20 1

15 19 1

15 18 1

14 21 1

14 17 1

13 21 1

13 15 1

12 16 1

12 22 1

12 23 1

11 15 1

11 24 1

10 14 1

10 13 1

10 22 1

10 23 1

9 16 1

9 14 1

9 13 1

9 21 1

8 15 1

8 17 1

8 21 1

7 11 1

7 14 1

7 13 1

7 10 1

7 19 1

7 18 1

6 17 1

6 12 1

6 9 1

6 15 1

5 16 1

5 19 1

5 18 1

5 8 1

20 4 1

4 7 1

4 15 1

3 19 1

3 74 1

3 16 1

3 18 1

3 6 1

2 19 1

2 4 1

2 77 1

1 17 1

1 75 1

1 76 1

1 5 1

[ angles ]

; ai aj ak funct theta cth

83 77 84 1 1.0835e+02 3.2995e+02

81 76 82 1 1.0835e+02 3.2995e+02

79 77 83 1 1.0968e+02 3.9497e+02

79 77 84 1 1.0968e+02 3.9497e+02

77 76 81 1 1.1005e+02 3.8802e+02

77 76 82 1 1.1005e+02 3.8802e+02

76 77 83 1 1.1005e+02 3.8802e+02

76 77 84 1 1.1005e+02 3.8802e+02

74 76 81 1 1.0968e+02 3.9497e+02

74 76 82 1 1.0968e+02 3.9497e+02

72 28 73 1 1.0835e+02 3.2995e+02

71 28 72 1 1.0835e+02 3.2995e+02

71 28 73 1 1.0835e+02 3.2995e+02

69 27 70 1 1.0835e+02 3.2995e+02

68 27 69 1 1.0835e+02 3.2995e+02

68 27 70 1 1.0835e+02 3.2995e+02

65 25 66 1 1.0835e+02 3.2995e+02

63 24 64 1 1.0835e+02 3.2995e+02

61 23 62 1 1.0835e+02 3.2995e+02

59 22 60 1 1.0835e+02 3.2995e+02

58 22 59 1 1.0835e+02 3.2995e+02

58 22 60 1 1.0835e+02 3.2995e+02

55 20 56 1 1.0835e+02 3.2995e+02

53 19 54 1 1.0835e+02 3.2995e+02

51 18 52 1 1.0835e+02 3.2995e+02

50 18 51 1 1.0835e+02 3.2995e+02

50 18 52 1 1.0835e+02 3.2995e+02

47 15 48 1 1.0835e+02 3.2995e+02

45 14 46 1 1.0835e+02 3.2995e+02

43 13 44 1 1.0835e+02 3.2995e+02

42 13 43 1 1.0835e+02 3.2995e+02

42 13 44 1 1.0835e+02 3.2995e+02

39 10 40 1 1.0835e+02 3.2995e+02

37 9 38 1 1.0835e+02 3.2995e+02

33 6 34 1 1.0835e+02 3.2995e+02

30 3 31 1 1.0835e+02 3.2995e+02

28 26 67 1 1.1005e+02 3.8802e+02

27 26 67 1 1.1005e+02 3.8802e+02

26 25 66 1 1.1005e+02 3.8802e+02

26 25 65 1 1.1005e+02 3.8802e+02

26 27 68 1 1.1005e+02 3.8802e+02

26 27 69 1 1.1005e+02 3.8802e+02

26 27 70 1 1.1005e+02 3.8802e+02

26 28 71 1 1.1005e+02 3.8802e+02

26 28 72 1 1.1005e+02 3.8802e+02

26 28 73 1 1.1005e+02 3.8802e+02

25 24 63 1 1.1005e+02 3.8802e+02

25 24 64 1 1.1005e+02 3.8802e+02

25 26 67 1 1.1005e+02 3.8802e+02

24 23 61 1 1.1005e+02 3.8802e+02

24 23 62 1 1.1005e+02 3.8802e+02

24 25 66 1 1.1005e+02 3.8802e+02

24 25 65 1 1.1005e+02 3.8802e+02

23 21 57 1 1.1005e+02 3.8802e+02

23 24 63 1 1.1005e+02 3.8802e+02

23 24 64 1 1.1005e+02 3.8802e+02

22 21 57 1 1.1005e+02 3.8802e+02

21 11 41 1 1.1005e+02 3.8802e+02

21 22 58 1 1.1005e+02 3.8802e+02

21 22 59 1 1.1005e+02 3.8802e+02

21 22 60 1 1.1005e+02 3.8802e+02

21 23 61 1 1.1005e+02 3.8802e+02

21 23 62 1 1.1005e+02 3.8802e+02

20 1 29 1 1.1005e+02 3.8802e+02

20 19 53 1 1.1005e+02 3.8802e+02

20 19 54 1 1.1005e+02 3.8802e+02

19 20 55 1 1.1005e+02 3.8802e+02

19 20 56 1 1.1005e+02 3.8802e+02

17 16 49 1 1.1005e+02 3.8802e+02

17 19 53 1 1.1005e+02 3.8802e+02

17 19 54 1 1.1005e+02 3.8802e+02

17 18 50 1 1.1005e+02 3.8802e+02

17 18 51 1 1.1005e+02 3.8802e+02

17 18 52 1 1.1005e+02 3.8802e+02

16 7 35 1 1.1005e+02 3.8802e+02

16 15 47 1 1.1005e+02 3.8802e+02

16 15 48 1 1.1005e+02 3.8802e+02

15 14 45 1 1.1005e+02 3.8802e+02

15 14 46 1 1.1005e+02 3.8802e+02

15 16 49 1 1.1005e+02 3.8802e+02

14 15 47 1 1.1005e+02 3.8802e+02

14 15 48 1 1.1005e+02 3.8802e+02

12 8 36 1 1.1005e+02 3.8802e+02

12 11 41 1 1.1005e+02 3.8802e+02

12 14 45 1 1.1005e+02 3.8802e+02

12 14 46 1 1.1005e+02 3.8802e+02

12 13 42 1 1.1005e+02 3.8802e+02

12 13 43 1 1.1005e+02 3.8802e+02

12 13 44 1 1.1005e+02 3.8802e+02

11 10 39 1 1.1005e+02 3.8802e+02

11 10 40 1 1.1005e+02 3.8802e+02

11 21 57 1 1.1005e+02 3.8802e+02

10 9 37 1 1.1005e+02 3.8802e+02

10 9 38 1 1.1005e+02 3.8802e+02

10 11 41 1 1.1005e+02 3.8802e+02

9 8 36 1 1.1005e+02 3.8802e+02

9 10 39 1 1.1005e+02 3.8802e+02

9 10 40 1 1.1005e+02 3.8802e+02

8 7 35 1 1.1005e+02 3.8802e+02

8 9 37 1 1.1005e+02 3.8802e+02

8 9 38 1 1.1005e+02 3.8802e+02

7 6 34 1 1.1005e+02 3.8802e+02

7 6 33 1 1.1005e+02 3.8802e+02

7 8 36 1 1.1005e+02 3.8802e+02

7 16 49 1 1.1005e+02 3.8802e+02

6 5 32 1 1.2000e+02 3.7782e+02

6 7 35 1 1.1005e+02 3.8802e+02

5 6 34 1 1.1049e+02 3.9355e+02

5 6 33 1 1.1049e+02 3.9355e+02

4 3 30 1 1.1049e+02 3.9355e+02

4 3 31 1 1.1049e+02 3.9355e+02

4 5 32 1 1.1970e+02 4.2091e+02

3 1 29 1 1.1005e+02 3.8802e+02

2 1 29 1 1.0870e+02 4.2568e+02

1 20 55 1 1.1005e+02 3.8802e+02

1 20 56 1 1.1005e+02 3.8802e+02

1 3 30 1 1.1005e+02 3.8802e+02

1 3 31 1 1.1005e+02 3.8802e+02

78 79 80 1 1.2288e+02 6.4752e+02

77 79 78 1 1.2311e+02 5.6928e+02

77 79 80 1 1.1220e+02 5.8442e+02

76 77 79 1 1.1053e+02 5.3379e+02

75 74 76 1 1.2311e+02 5.6928e+02

74 76 77 1 1.1053e+02 5.3379e+02

27 26 28 1 1.1063e+02 5.2894e+02

25 26 27 1 1.1063e+02 5.2894e+02

25 26 28 1 1.1063e+02 5.2894e+02

24 25 26 1 1.1063e+02 5.2894e+02

23 24 25 1 1.1063e+02 5.2894e+02

22 21 23 1 1.1063e+02 5.2894e+02

21 23 24 1 1.1063e+02 5.2894e+02

18 17 19 1 1.1063e+02 5.2894e+02

17 19 20 1 1.1063e+02 5.2894e+02

16 17 19 1 1.1063e+02 5.2894e+02

16 17 18 1 1.1063e+02 5.2894e+02

15 16 17 1 1.1063e+02 5.2894e+02

14 15 16 1 1.1063e+02 5.2894e+02

13 12 14 1 1.1063e+02 5.2894e+02

12 11 21 1 1.1063e+02 5.2894e+02

12 14 15 1 1.1063e+02 5.2894e+02

11 12 14 1 1.1063e+02 5.2894e+02

11 12 13 1 1.1063e+02 5.2894e+02

11 21 22 1 1.1063e+02 5.2894e+02

11 21 23 1 1.1063e+02 5.2894e+02

10 11 12 1 1.1063e+02 5.2894e+02

10 11 21 1 1.1063e+02 5.2894e+02

9 8 12 1 1.1063e+02 5.2894e+02

9 10 11 1 1.1063e+02 5.2894e+02

8 7 16 1 1.1063e+02 5.2894e+02

8 12 11 1 1.1063e+02 5.2894e+02

8 12 14 1 1.1063e+02 5.2894e+02

8 12 13 1 1.1063e+02 5.2894e+02

8 9 10 1 1.1063e+02 5.2894e+02

7 8 12 1 1.1063e+02 5.2894e+02

7 8 9 1 1.1063e+02 5.2894e+02

7 16 15 1 1.1063e+02 5.2894e+02

7 16 17 1 1.1063e+02 5.2894e+02

6 7 8 1 1.1063e+02 5.2894e+02

6 7 16 1 1.1063e+02 5.2894e+02

5 4 17 1 1.2342e+02 5.3831e+02

5 6 7 1 1.1144e+02 5.3162e+02

4 17 16 1 1.1144e+02 5.3162e+02

4 17 19 1 1.1144e+02 5.3162e+02

4 17 18 1 1.1144e+02 5.3162e+02

4 5 6 1 1.2342e+02 5.3831e+02

3 1 20 1 1.1063e+02 5.2894e+02

3 4 17 1 1.1652e+02 5.2467e+02

3 4 5 1 1.2342e+02 5.3831e+02

2 1 20 1 1.0842e+02 5.6718e+02

2 1 3 1 1.0842e+02 5.6718e+02

2 74 75 1 1.2333e+02 6.3538e+02

2 74 76 1 1.1196e+02 5.7957e+02

1 20 19 1 1.1063e+02 5.2894e+02

1 2 74 1 1.1514e+02 5.3246e+02

1 3 4 1 1.1144e+02 5.3162e+02

[ dihedrals ]

;i j k l func C0 ... C5

82 76 77 83 3 0.62760 1.88280 0.00000 -2.51040 0.00000 0.00000 ;

82 76 77 84 3 0.62760 1.88280 0.00000 -2.51040 0.00000 0.00000 ;

81 76 77 83 3 0.62760 1.88280 0.00000 -2.51040 0.00000 0.00000 ;

81 76 77 84 3 0.62760 1.88280 0.00000 -2.51040 0.00000 0.00000 ;

80 79 77 83 3 0.00000 0.00000 0.00000 0.00000 0.00000 0.00000 ;

80 79 77 84 3 0.00000 0.00000 0.00000 0.00000 0.00000 0.00000 ;

79 77 76 81 3 0.65270 1.95811 0.00000 -2.61082 0.00000 0.00000 ;

79 77 76 82 3 0.65270 1.95811 0.00000 -2.61082 0.00000 0.00000 ;

78 79 77 83 3 3.68192 -4.35136 0.00000 1.33888 0.00000 0.00000 ;

78 79 77 84 3 3.68192 -4.35136 0.00000 1.33888 0.00000 0.00000 ;

75 74 76 81 3 3.68192 -4.35136 0.00000 1.33888 0.00000 0.00000 ;

75 74 76 82 3 3.68192 -4.35136 0.00000 1.33888 0.00000 0.00000 ;

74 76 77 83 3 0.65270 1.95811 0.00000 -2.61082 0.00000 0.00000 ;

74 76 77 84 3 0.65270 1.95811 0.00000 -2.61082 0.00000 0.00000 ;

67 26 27 68 3 0.62760 1.88280 0.00000 -2.51040 0.00000 0.00000 ;

67 26 27 69 3 0.62760 1.88280 0.00000 -2.51040 0.00000 0.00000 ;

67 26 27 70 3 0.62760 1.88280 0.00000 -2.51040 0.00000 0.00000 ;

67 26 28 71 3 0.62760 1.88280 0.00000 -2.51040 0.00000 0.00000 ;

67 26 28 72 3 0.62760 1.88280 0.00000 -2.51040 0.00000 0.00000 ;

67 26 28 73 3 0.62760 1.88280 0.00000 -2.51040 0.00000 0.00000 ;

66 25 26 67 3 0.62760 1.88280 0.00000 -2.51040 0.00000 0.00000 ;

65 25 26 67 3 0.62760 1.88280 0.00000 -2.51040 0.00000 0.00000 ;

64 24 25 66 3 0.62760 1.88280 0.00000 -2.51040 0.00000 0.00000 ;

64 24 25 65 3 0.62760 1.88280 0.00000 -2.51040 0.00000 0.00000 ;

63 24 25 66 3 0.62760 1.88280 0.00000 -2.51040 0.00000 0.00000 ;

63 24 25 65 3 0.62760 1.88280 0.00000 -2.51040 0.00000 0.00000 ;

62 23 24 63 3 0.62760 1.88280 0.00000 -2.51040 0.00000 0.00000 ;

62 23 24 64 3 0.62760 1.88280 0.00000 -2.51040 0.00000 0.00000 ;

61 23 24 63 3 0.62760 1.88280 0.00000 -2.51040 0.00000 0.00000 ;

61 23 24 64 3 0.62760 1.88280 0.00000 -2.51040 0.00000 0.00000 ;

57 21 22 58 3 0.62760 1.88280 0.00000 -2.51040 0.00000 0.00000 ;

57 21 22 59 3 0.62760 1.88280 0.00000 -2.51040 0.00000 0.00000 ;

57 21 22 60 3 0.62760 1.88280 0.00000 -2.51040 0.00000 0.00000 ;

57 21 23 61 3 0.62760 1.88280 0.00000 -2.51040 0.00000 0.00000 ;

57 21 23 62 3 0.62760 1.88280 0.00000 -2.51040 0.00000 0.00000 ;

54 19 20 55 3 0.62760 1.88280 0.00000 -2.51040 0.00000 0.00000 ;

54 19 20 56 3 0.62760 1.88280 0.00000 -2.51040 0.00000 0.00000 ;

53 19 20 55 3 0.62760 1.88280 0.00000 -2.51040 0.00000 0.00000 ;

53 19 20 56 3 0.62760 1.88280 0.00000 -2.51040 0.00000 0.00000 ;

48 15 16 49 3 0.62760 1.88280 0.00000 -2.51040 0.00000 0.00000 ;

47 15 16 49 3 0.62760 1.88280 0.00000 -2.51040 0.00000 0.00000 ;

46 14 15 47 3 0.62760 1.88280 0.00000 -2.51040 0.00000 0.00000 ;

46 14 15 48 3 0.62760 1.88280 0.00000 -2.51040 0.00000 0.00000 ;

45 14 15 47 3 0.62760 1.88280 0.00000 -2.51040 0.00000 0.00000 ;

45 14 15 48 3 0.62760 1.88280 0.00000 -2.51040 0.00000 0.00000 ;

41 11 21 57 3 0.62760 1.88280 0.00000 -2.51040 0.00000 0.00000 ;

40 10 11 41 3 0.62760 1.88280 0.00000 -2.51040 0.00000 0.00000 ;

39 10 11 41 3 0.62760 1.88280 0.00000 -2.51040 0.00000 0.00000 ;

38 9 10 39 3 0.62760 1.88280 0.00000 -2.51040 0.00000 0.00000 ;

38 9 10 40 3 0.62760 1.88280 0.00000 -2.51040 0.00000 0.00000 ;

37 9 10 39 3 0.62760 1.88280 0.00000 -2.51040 0.00000 0.00000 ;

37 9 10 40 3 0.62760 1.88280 0.00000 -2.51040 0.00000 0.00000 ;

36 8 9 37 3 0.62760 1.88280 0.00000 -2.51040 0.00000 0.00000 ;

36 8 9 38 3 0.62760 1.88280 0.00000 -2.51040 0.00000 0.00000 ;

35 7 8 36 3 0.62760 1.88280 0.00000 -2.51040 0.00000 0.00000 ;

35 7 16 49 3 0.62760 1.88280 0.00000 -2.51040 0.00000 0.00000 ;

34 6 7 35 3 0.62760 1.88280 0.00000 -2.51040 0.00000 0.00000 ;

33 6 7 35 3 0.62760 1.88280 0.00000 -2.51040 0.00000 0.00000 ;

32 5 6 34 3 0.00000 0.00000 0.00000 0.00000 0.00000 0.00000 ;

32 5 6 33 3 0.00000 0.00000 0.00000 0.00000 0.00000 0.00000 ;

29 1 20 55 3 0.62760 1.88280 0.00000 -2.51040 0.00000 0.00000 ;

29 1 20 56 3 0.62760 1.88280 0.00000 -2.51040 0.00000 0.00000 ;

29 1 2 74 3 1.60247 4.80742 0.00000 -6.40989 0.00000 0.00000 ;

29 1 3 30 3 0.62760 1.88280 0.00000 -2.51040 0.00000 0.00000 ;

29 1 3 31 3 0.62760 1.88280 0.00000 -2.51040 0.00000 0.00000 ;

28 26 25 66 3 0.66944 2.00832 0.00000 -2.67776 0.00000 0.00000 ;

28 26 25 65 3 0.66944 2.00832 0.00000 -2.67776 0.00000 0.00000 ;

28 26 27 68 3 0.66944 2.00832 0.00000 -2.67776 0.00000 0.00000 ;

28 26 27 69 3 0.66944 2.00832 0.00000 -2.67776 0.00000 0.00000 ;

28 26 27 70 3 0.66944 2.00832 0.00000 -2.67776 0.00000 0.00000 ;

27 26 25 66 3 0.66944 2.00832 0.00000 -2.67776 0.00000 0.00000 ;

27 26 25 65 3 0.66944 2.00832 0.00000 -2.67776 0.00000 0.00000 ;

27 26 28 71 3 0.66944 2.00832 0.00000 -2.67776 0.00000 0.00000 ;

27 26 28 72 3 0.66944 2.00832 0.00000 -2.67776 0.00000 0.00000 ;

27 26 28 73 3 0.66944 2.00832 0.00000 -2.67776 0.00000 0.00000 ;

26 25 24 63 3 0.66944 2.00832 0.00000 -2.67776 0.00000 0.00000 ;

26 25 24 64 3 0.66944 2.00832 0.00000 -2.67776 0.00000 0.00000 ;

25 24 23 61 3 0.66944 2.00832 0.00000 -2.67776 0.00000 0.00000 ;

25 24 23 62 3 0.66944 2.00832 0.00000 -2.67776 0.00000 0.00000 ;

25 26 27 68 3 0.66944 2.00832 0.00000 -2.67776 0.00000 0.00000 ;

25 26 27 69 3 0.66944 2.00832 0.00000 -2.67776 0.00000 0.00000 ;

25 26 27 70 3 0.66944 2.00832 0.00000 -2.67776 0.00000 0.00000 ;

25 26 28 71 3 0.66944 2.00832 0.00000 -2.67776 0.00000 0.00000 ;

25 26 28 72 3 0.66944 2.00832 0.00000 -2.67776 0.00000 0.00000 ;

25 26 28 73 3 0.66944 2.00832 0.00000 -2.67776 0.00000 0.00000 ;

24 23 21 57 3 0.66944 2.00832 0.00000 -2.67776 0.00000 0.00000 ;

24 25 26 67 3 0.66944 2.00832 0.00000 -2.67776 0.00000 0.00000 ;

23 21 11 41 3 0.66944 2.00832 0.00000 -2.67776 0.00000 0.00000 ;

23 21 22 58 3 0.66944 2.00832 0.00000 -2.67776 0.00000 0.00000 ;

23 21 22 59 3 0.66944 2.00832 0.00000 -2.67776 0.00000 0.00000 ;

23 21 22 60 3 0.66944 2.00832 0.00000 -2.67776 0.00000 0.00000 ;

23 24 25 66 3 0.66944 2.00832 0.00000 -2.67776 0.00000 0.00000 ;

23 24 25 65 3 0.66944 2.00832 0.00000 -2.67776 0.00000 0.00000 ;

22 21 11 41 3 0.66944 2.00832 0.00000 -2.67776 0.00000 0.00000 ;

22 21 23 61 3 0.66944 2.00832 0.00000 -2.67776 0.00000 0.00000 ;

22 21 23 62 3 0.66944 2.00832 0.00000 -2.67776 0.00000 0.00000 ;

21 11 10 39 3 0.66944 2.00832 0.00000 -2.67776 0.00000 0.00000 ;

21 11 10 40 3 0.66944 2.00832 0.00000 -2.67776 0.00000 0.00000 ;

21 23 24 63 3 0.66944 2.00832 0.00000 -2.67776 0.00000 0.00000 ;

21 23 24 64 3 0.66944 2.00832 0.00000 -2.67776 0.00000 0.00000 ;

20 1 3 30 3 0.66944 2.00832 0.00000 -2.67776 0.00000 0.00000 ;

20 1 3 31 3 0.66944 2.00832 0.00000 -2.67776 0.00000 0.00000 ;

19 17 16 49 3 0.66944 2.00832 0.00000 -2.67776 0.00000 0.00000 ;

19 17 18 50 3 0.66944 2.00832 0.00000 -2.67776 0.00000 0.00000 ;

19 17 18 51 3 0.66944 2.00832 0.00000 -2.67776 0.00000 0.00000 ;

19 17 18 52 3 0.66944 2.00832 0.00000 -2.67776 0.00000 0.00000 ;

29 1 20 19 3 0.66944 2.00832 0.00000 -2.67776 0.00000 0.00000 ;

18 17 16 49 3 0.66944 2.00832 0.00000 -2.67776 0.00000 0.00000 ;

18 17 19 53 3 0.66944 2.00832 0.00000 -2.67776 0.00000 0.00000 ;

18 17 19 54 3 0.66944 2.00832 0.00000 -2.67776 0.00000 0.00000 ;

17 4 3 30 3 0.00000 0.00000 0.00000 0.00000 0.00000 0.00000 ;

17 4 3 31 3 0.00000 0.00000 0.00000 0.00000 0.00000 0.00000 ;

17 4 5 32 3 55.64720 0.00000 -55.64720 0.00000 0.00000 0.00000 ;

17 16 7 35 3 0.66944 2.00832 0.00000 -2.67776 0.00000 0.00000 ;

17 16 15 47 3 0.66944 2.00832 0.00000 -2.67776 0.00000 0.00000 ;

17 16 15 48 3 0.66944 2.00832 0.00000 -2.67776 0.00000 0.00000 ;

17 19 20 55 3 0.66944 2.00832 0.00000 -2.67776 0.00000 0.00000 ;

17 19 20 56 3 0.66944 2.00832 0.00000 -2.67776 0.00000 0.00000 ;

16 7 6 34 3 0.66944 2.00832 0.00000 -2.67776 0.00000 0.00000 ;

16 7 6 33 3 0.66944 2.00832 0.00000 -2.67776 0.00000 0.00000 ;

16 7 8 36 3 0.66944 2.00832 0.00000 -2.67776 0.00000 0.00000 ;

16 15 14 45 3 0.66944 2.00832 0.00000 -2.67776 0.00000 0.00000 ;

16 15 14 46 3 0.66944 2.00832 0.00000 -2.67776 0.00000 0.00000 ;

16 17 19 53 3 0.66944 2.00832 0.00000 -2.67776 0.00000 0.00000 ;

16 17 19 54 3 0.66944 2.00832 0.00000 -2.67776 0.00000 0.00000 ;

16 17 18 50 3 0.66944 2.00832 0.00000 -2.67776 0.00000 0.00000 ;

16 17 18 51 3 0.66944 2.00832 0.00000 -2.67776 0.00000 0.00000 ;

16 17 18 52 3 0.66944 2.00832 0.00000 -2.67776 0.00000 0.00000 ;

15 16 7 35 3 0.66944 2.00832 0.00000 -2.67776 0.00000 0.00000 ;

14 12 8 36 3 0.66944 2.00832 0.00000 -2.67776 0.00000 0.00000 ;

14 12 11 41 3 0.66944 2.00832 0.00000 -2.67776 0.00000 0.00000 ;

14 12 13 42 3 0.66944 2.00832 0.00000 -2.67776 0.00000 0.00000 ;

14 12 13 43 3 0.66944 2.00832 0.00000 -2.67776 0.00000 0.00000 ;

14 12 13 44 3 0.66944 2.00832 0.00000 -2.67776 0.00000 0.00000 ;

14 15 16 49 3 0.66944 2.00832 0.00000 -2.67776 0.00000 0.00000 ;

13 12 8 36 3 0.66944 2.00832 0.00000 -2.67776 0.00000 0.00000 ;

13 12 11 41 3 0.66944 2.00832 0.00000 -2.67776 0.00000 0.00000 ;

13 12 14 45 3 0.66944 2.00832 0.00000 -2.67776 0.00000 0.00000 ;

13 12 14 46 3 0.66944 2.00832 0.00000 -2.67776 0.00000 0.00000 ;

12 8 7 35 3 0.66944 2.00832 0.00000 -2.67776 0.00000 0.00000 ;

12 8 9 37 3 0.66944 2.00832 0.00000 -2.67776 0.00000 0.00000 ;

12 8 9 38 3 0.66944 2.00832 0.00000 -2.67776 0.00000 0.00000 ;

12 11 10 39 3 0.66944 2.00832 0.00000 -2.67776 0.00000 0.00000 ;

12 11 10 40 3 0.66944 2.00832 0.00000 -2.67776 0.00000 0.00000 ;

12 11 21 57 3 0.66944 2.00832 0.00000 -2.67776 0.00000 0.00000 ;

12 14 15 47 3 0.66944 2.00832 0.00000 -2.67776 0.00000 0.00000 ;

12 14 15 48 3 0.66944 2.00832 0.00000 -2.67776 0.00000 0.00000 ;

11 10 9 37 3 0.66944 2.00832 0.00000 -2.67776 0.00000 0.00000 ;

11 10 9 38 3 0.66944 2.00832 0.00000 -2.67776 0.00000 0.00000 ;

11 12 8 36 3 0.66944 2.00832 0.00000 -2.67776 0.00000 0.00000 ;

11 12 14 45 3 0.66944 2.00832 0.00000 -2.67776 0.00000 0.00000 ;

11 12 14 46 3 0.66944 2.00832 0.00000 -2.67776 0.00000 0.00000 ;

11 12 13 42 3 0.66944 2.00832 0.00000 -2.67776 0.00000 0.00000 ;

11 12 13 43 3 0.66944 2.00832 0.00000 -2.67776 0.00000 0.00000 ;

11 12 13 44 3 0.66944 2.00832 0.00000 -2.67776 0.00000 0.00000 ;

11 21 22 58 3 0.66944 2.00832 0.00000 -2.67776 0.00000 0.00000 ;

11 21 22 59 3 0.66944 2.00832 0.00000 -2.67776 0.00000 0.00000 ;

11 21 22 60 3 0.66944 2.00832 0.00000 -2.67776 0.00000 0.00000 ;

11 21 23 61 3 0.66944 2.00832 0.00000 -2.67776 0.00000 0.00000 ;

11 21 23 62 3 0.66944 2.00832 0.00000 -2.67776 0.00000 0.00000 ;

10 9 8 36 3 0.66944 2.00832 0.00000 -2.67776 0.00000 0.00000 ;

10 11 21 57 3 0.66944 2.00832 0.00000 -2.67776 0.00000 0.00000 ;

9 8 7 35 3 0.66944 2.00832 0.00000 -2.67776 0.00000 0.00000 ;

9 10 11 41 3 0.66944 2.00832 0.00000 -2.67776 0.00000 0.00000 ;

8 7 6 34 3 0.66944 2.00832 0.00000 -2.67776 0.00000 0.00000 ;

8 7 6 33 3 0.66944 2.00832 0.00000 -2.67776 0.00000 0.00000 ;

8 7 16 49 3 0.66944 2.00832 0.00000 -2.67776 0.00000 0.00000 ;

8 12 11 41 3 0.66944 2.00832 0.00000 -2.67776 0.00000 0.00000 ;

8 12 14 45 3 0.66944 2.00832 0.00000 -2.67776 0.00000 0.00000 ;

8 12 14 46 3 0.66944 2.00832 0.00000 -2.67776 0.00000 0.00000 ;

8 12 13 42 3 0.66944 2.00832 0.00000 -2.67776 0.00000 0.00000 ;

8 12 13 43 3 0.66944 2.00832 0.00000 -2.67776 0.00000 0.00000 ;

8 12 13 44 3 0.66944 2.00832 0.00000 -2.67776 0.00000 0.00000 ;

8 9 10 39 3 0.66944 2.00832 0.00000 -2.67776 0.00000 0.00000 ;

8 9 10 40 3 0.66944 2.00832 0.00000 -2.67776 0.00000 0.00000 ;

7 6 5 32 3 0.00000 0.00000 0.00000 0.00000 0.00000 0.00000 ;

7 8 9 37 3 0.66944 2.00832 0.00000 -2.67776 0.00000 0.00000 ;

7 8 9 38 3 0.66944 2.00832 0.00000 -2.67776 0.00000 0.00000 ;

7 16 15 47 3 0.66944 2.00832 0.00000 -2.67776 0.00000 0.00000 ;

7 16 15 48 3 0.66944 2.00832 0.00000 -2.67776 0.00000 0.00000 ;

6 7 8 36 3 0.66944 2.00832 0.00000 -2.67776 0.00000 0.00000 ;

6 7 16 49 3 0.66944 2.00832 0.00000 -2.67776 0.00000 0.00000 ;

5 4 3 30 3 6.40152 -9.58136 0.00000 6.35968 0.00000 0.00000 ;

5 4 3 31 3 6.40152 -9.58136 0.00000 6.35968 0.00000 0.00000 ;

5 6 7 35 3 0.65270 1.95811 0.00000 -2.61082 0.00000 0.00000 ;

29 1 3 4 3 0.65270 1.95811 0.00000 -2.61082 0.00000 0.00000 ;

4 17 16 49 3 0.65270 1.95811 0.00000 -2.61082 0.00000 0.00000 ;

4 17 19 53 3 0.65270 1.95811 0.00000 -2.61082 0.00000 0.00000 ;

4 17 19 54 3 0.65270 1.95811 0.00000 -2.61082 0.00000 0.00000 ;

4 17 18 50 3 0.65270 1.95811 0.00000 -2.61082 0.00000 0.00000 ;

4 17 18 51 3 0.65270 1.95811 0.00000 -2.61082 0.00000 0.00000 ;

4 17 18 52 3 0.65270 1.95811 0.00000 -2.61082 0.00000 0.00000 ;

4 5 6 34 3 6.40152 -9.58136 0.00000 6.35968 0.00000 0.00000 ;

4 5 6 33 3 6.40152 -9.58136 0.00000 6.35968 0.00000 0.00000 ;

3 1 20 55 3 0.66944 2.00832 0.00000 -2.67776 0.00000 0.00000 ;

3 1 20 56 3 0.66944 2.00832 0.00000 -2.67776 0.00000 0.00000 ;

3 4 5 32 3 55.64720 0.00000 -55.64720 0.00000 0.00000 0.00000 ;

2 1 20 55 3 1.04600 -1.04600 0.00000 0.00000 0.00000 0.00000 ;

2 1 20 56 3 1.04600 -1.04600 0.00000 0.00000 0.00000 0.00000 ;

2 1 3 30 3 1.04600 -1.04600 0.00000 0.00000 0.00000 0.00000 ;

2 1 3 31 3 1.04600 -1.04600 0.00000 0.00000 0.00000 0.00000 ;

2 74 76 81 3 0.00000 0.00000 0.00000 0.00000 0.00000 0.00000 ;

2 74 76 82 3 0.00000 0.00000 0.00000 0.00000 0.00000 0.00000 ;

1 20 19 53 3 0.66944 2.00832 0.00000 -2.67776 0.00000 0.00000 ;

1 20 19 54 3 0.66944 2.00832 0.00000 -2.67776 0.00000 0.00000 ;

4 6 5 32 3 9.20480 0.00000 -9.20480 0.00000 0.00000 0.00000 ;

76 77 79 78 3 0.00000 0.00000 0.00000 0.00000 0.00000 0.00000 ;

76 77 79 80 3 0.00000 0.00000 0.00000 0.00000 0.00000 0.00000 ;

75 74 76 77 3 0.00000 0.00000 0.00000 0.00000 0.00000 0.00000 ;

74 76 77 79 3 0.65270 1.95811 0.00000 -2.61082 0.00000 0.00000 ;

24 25 26 27 3 3.68192 3.09616 -2.09200 -3.01248 0.00000 0.00000 ;

24 25 26 28 3 3.68192 3.09616 -2.09200 -3.01248 0.00000 0.00000 ;

23 24 25 26 3 3.68192 3.09616 -2.09200 -3.01248 0.00000 0.00000 ;

22 21 23 24 3 3.68192 3.09616 -2.09200 -3.01248 0.00000 0.00000 ;

21 23 24 25 3 3.68192 3.09616 -2.09200 -3.01248 0.00000 0.00000 ;

20 1 2 74 3 4.94967 8.15462 0.00000 -6.40989 0.00000 0.00000 ;

18 17 19 20 3 3.68192 3.09616 -2.09200 -3.01248 0.00000 0.00000 ;

16 17 19 20 3 3.68192 3.09616 -2.09200 -3.01248 0.00000 0.00000 ;

15 16 17 19 3 3.68192 3.09616 -2.09200 -3.01248 0.00000 0.00000 ;

15 16 17 18 3 3.68192 3.09616 -2.09200 -3.01248 0.00000 0.00000 ;

14 12 11 21 3 3.68192 3.09616 -2.09200 -3.01248 0.00000 0.00000 ;

14 15 16 17 3 3.68192 3.09616 -2.09200 -3.01248 0.00000 0.00000 ;

13 12 11 21 3 3.68192 3.09616 -2.09200 -3.01248 0.00000 0.00000 ;

13 12 14 15 3 3.68192 3.09616 -2.09200 -3.01248 0.00000 0.00000 ;

12 8 7 16 3 3.68192 3.09616 -2.09200 -3.01248 0.00000 0.00000 ;

12 11 21 22 3 3.68192 3.09616 -2.09200 -3.01248 0.00000 0.00000 ;

12 11 21 23 3 3.68192 3.09616 -2.09200 -3.01248 0.00000 0.00000 ;

12 14 15 16 3 3.68192 3.09616 -2.09200 -3.01248 0.00000 0.00000 ;

11 12 14 15 3 3.68192 3.09616 -2.09200 -3.01248 0.00000 0.00000 ;

11 21 23 24 3 3.68192 3.09616 -2.09200 -3.01248 0.00000 0.00000 ;

10 9 8 12 3 3.68192 3.09616 -2.09200 -3.01248 0.00000 0.00000 ;

10 11 12 14 3 3.68192 3.09616 -2.09200 -3.01248 0.00000 0.00000 ;

10 11 12 13 3 3.68192 3.09616 -2.09200 -3.01248 0.00000 0.00000 ;

10 11 21 22 3 3.68192 3.09616 -2.09200 -3.01248 0.00000 0.00000 ;

10 11 21 23 3 3.68192 3.09616 -2.09200 -3.01248 0.00000 0.00000 ;

9 8 7 16 3 3.68192 3.09616 -2.09200 -3.01248 0.00000 0.00000 ;

9 8 12 11 3 3.68192 3.09616 -2.09200 -3.01248 0.00000 0.00000 ;

9 8 12 14 3 3.68192 3.09616 -2.09200 -3.01248 0.00000 0.00000 ;

9 8 12 13 3 3.68192 3.09616 -2.09200 -3.01248 0.00000 0.00000 ;

9 10 11 12 3 3.68192 3.09616 -2.09200 -3.01248 0.00000 0.00000 ;

9 10 11 21 3 3.68192 3.09616 -2.09200 -3.01248 0.00000 0.00000 ;

8 7 16 15 3 3.68192 3.09616 -2.09200 -3.01248 0.00000 0.00000 ;

8 7 16 17 3 3.68192 3.09616 -2.09200 -3.01248 0.00000 0.00000 ;

8 12 11 10 3 3.68192 3.09616 -2.09200 -3.01248 0.00000 0.00000 ;

8 12 11 21 3 3.68192 3.09616 -2.09200 -3.01248 0.00000 0.00000 ;

8 12 14 15 3 3.68192 3.09616 -2.09200 -3.01248 0.00000 0.00000 ;

8 9 10 11 3 3.68192 3.09616 -2.09200 -3.01248 0.00000 0.00000 ;

7 8 12 11 3 3.68192 3.09616 -2.09200 -3.01248 0.00000 0.00000 ;

7 8 12 14 3 3.68192 3.09616 -2.09200 -3.01248 0.00000 0.00000 ;

7 8 12 13 3 3.68192 3.09616 -2.09200 -3.01248 0.00000 0.00000 ;

7 8 9 10 3 3.68192 3.09616 -2.09200 -3.01248 0.00000 0.00000 ;

7 16 15 14 3 3.68192 3.09616 -2.09200 -3.01248 0.00000 0.00000 ;

7 16 17 19 3 3.68192 3.09616 -2.09200 -3.01248 0.00000 0.00000 ;

7 16 17 18 3 3.68192 3.09616 -2.09200 -3.01248 0.00000 0.00000 ;

6 5 4 17 3 63.59680 7.94960 -55.64720 0.00000 0.00000 0.00000 ;

6 7 8 12 3 3.68192 3.09616 -2.09200 -3.01248 0.00000 0.00000 ;

6 7 8 9 3 3.68192 3.09616 -2.09200 -3.01248 0.00000 0.00000 ;

6 7 16 15 3 3.68192 3.09616 -2.09200 -3.01248 0.00000 0.00000 ;

6 7 16 17 3 3.68192 3.09616 -2.09200 -3.01248 0.00000 0.00000 ;

5 4 17 16 3 0.00000 0.00000 0.00000 0.00000 0.00000 0.00000 ;

5 4 17 19 3 0.00000 0.00000 0.00000 0.00000 0.00000 0.00000 ;

5 4 17 18 3 0.00000 0.00000 0.00000 0.00000 0.00000 0.00000 ;

5 6 7 8 3 0.65270 1.95811 0.00000 -2.61082 0.00000 0.00000 ;

5 6 7 16 3 0.65270 1.95811 0.00000 -2.61082 0.00000 0.00000 ;

20 1 3 4 3 0.65270 1.95811 0.00000 -2.61082 0.00000 0.00000 ;

4 17 16 7 3 0.65270 1.95811 0.00000 -2.61082 0.00000 0.00000 ;

4 17 16 15 3 0.65270 1.95811 0.00000 -2.61082 0.00000 0.00000 ;

4 17 19 20 3 0.65270 1.95811 0.00000 -2.61082 0.00000 0.00000 ;

4 5 6 7 3 0.00000 0.00000 0.00000 0.00000 0.00000 0.00000 ;

3 1 20 19 3 3.68192 3.09616 -2.09200 -3.01248 0.00000 0.00000 ;

3 1 2 74 3 4.94967 8.15462 0.00000 -6.40989 0.00000 0.00000 ;

3 4 17 16 3 0.00000 0.00000 0.00000 0.00000 0.00000 0.00000 ;

3 4 17 19 3 0.00000 0.00000 0.00000 0.00000 0.00000 0.00000 ;

3 4 17 18 3 0.00000 0.00000 0.00000 0.00000 0.00000 0.00000 ;

3 4 5 6 3 63.59680 7.94960 -55.64720 0.00000 0.00000 0.00000 ;

2 1 20 19 3 0.65270 1.95811 0.00000 -2.61082 0.00000 0.00000 ;

2 1 3 4 3 0.65270 1.95811 0.00000 -2.61082 0.00000 0.00000 ;

2 74 76 77 3 0.00000 0.00000 0.00000 0.00000 0.00000 0.00000 ;

1 20 19 17 3 3.68192 3.09616 -2.09200 -3.01248 0.00000 0.00000 ;

1 2 74 75 3 28.45120 5.85760 -22.59360 0.00000 0.00000 0.00000 ;

1 2 74 76 3 22.59360 0.00000 -22.59360 0.00000 0.00000 0.00000 ;

1 3 4 17 3 0.00000 0.00000 0.00000 0.00000 0.00000 0.00000 ;

1 3 4 5 3 0.00000 0.00000 0.00000 0.00000 0.00000 0.00000 ;

77 78 79 80 3 9.20480 0.00000 -9.20480 0.00000 0.00000 0.00000 ;

76 75 74 2 3 9.20480 0.00000 -9.20480 0.00000 0.00000 0.00000 ;

5 3 4 17 3 9.20480 0.00000 -9.20480 0.00000 0.00000 0.00000 ;
